# MK4 Repositioning for IAHSP: Overcoming *In Vivo* Data Gaps through *In Silico* Refinement and *In Vitro* Validation

**DOI:** 10.1021/acschemneuro.5c00601

**Published:** 2026-02-09

**Authors:** Matteo Rossi Sebastiano, Antonio Vicidomini, Serena Francisco, Verdiana Pullano, Paola Defilippi, Gabriele Baj, Fabrizia Cesca, Giulia Caron, Giuseppe Ermondi

**Affiliations:** † 9314 University of Torino, Molecular Biotechnology and Health Sciences Department, Turin 10126, Italy; ‡ 9315 University of Trieste, Life Science Department, Trieste 34127, Italy; § University of Turin, Department of Neurosciences Rita Levi-Montalcini, Turin 10126, Italy; ∥ 9308 University of Padova, Department of Biomedical Sciences, Padova 35131, Italy

**Keywords:** IAHSP, ALSIN, MK4, mitochondria, drug repurposing, fibroblasts

## Abstract

Infantile-onset Ascending Hereditary Spastic Paralysis (IAHSP) is an ultrarare, autosomal recessive form of Hereditary Spastic Paraplegia (HSP), caused by mutations in the *ALS2* gene, which encodes the protein ALSIN. In a previous study, we proposed a personalized therapeutic strategy for an Italian IAHSP patient (AO), aiming to correct the aberrant function of the R1611W mutant ALSIN using Menatetrenone (MK4). While our results supported compassionate-use approval for a patient-specific therapeutic regimen, further investigation was needed to highlight the treatment’s benefits in the absence of tractable biophysical assays and *in vivo* models. In this respect, we first characterized MK4’s interaction with the mutation site through Molecular Dynamics simulations. Next, we established and characterized a skin fibroblast cell line derived from patient AO. We analyzed the expression and stability of the mutant ALSIN protein in AO’s fibroblasts and observed elevated oxidative stress levels. Using advanced microscopy and automated image analysis, we identified a characteristic mitochondrial phenotype associated with AO’s IAHSP. One specific morphological parameter of mitochondria (Mean Branch Diameter) accurately reflected the IAHSP phenotype and was selected as a cell marker. Treatment of IAHSP fibroblasts with MK4 highlighted the rescue of Mean Branch Diameter and ALSIN levels, supporting cellular efficacy. Overall, this work presents an approach that integrates computational and cell-based methodologies to overcome the data scarcity challenges of drug discovery in rare diseases. Our study provides a framework for preclinical, alternative drug discovery programs in monogenic rare disorders such as IAHSP.

## Graphical abstract

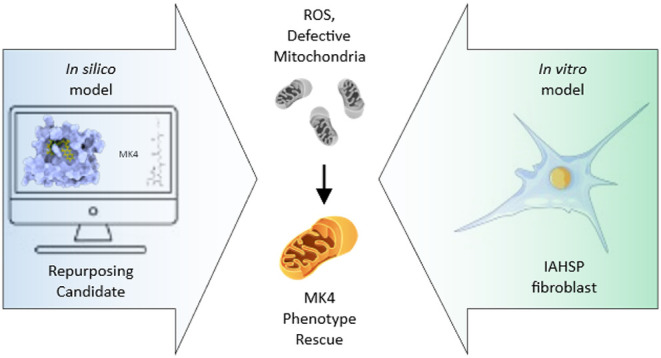

## Introduction

Hereditary Spastic Paraplegias (HSPs) are a group of poorly understood rare genetic diseases originating from mutations in different genes but bearing the common feature of limb stiffness and progressive degeneration.^1^ The challenge of developing treatments for rare/orphan diseases such as HSPs lies in a small patient population and limited research data, not attracting significant pharmaceutical investment. Furthermore, the variability of mutations calls for patient-specific approaches, making efficient and cost-effective research even more necessary than in widespread diseases.^2^ In this context, the traditional drug discovery workflow cannot always be fully applied since certain disease-relevant proteins, especially in their mutant forms, might be difficult to characterize experimentally. To meet these criteria and treat rare conditions, the whole drug discovery process should undergo a profound restructuring, characterized by the combination of different experimental approaches spanning from molecular modeling to *in vitro* experiments in a patient-focused manner.^2^

Infantile-onset Ascending Hereditary Spastic Paralysis (IAHSP, OMIM# 607225) is an autosomal recessive HSP occurring upon biallelic mutations in the gene *ALS2*. IAHSP is classified as ultrarare, affecting ca. 100 individuals worldwide.^3,4^ The protein encoded by the *ALS2* gene is ALSIN, a 180 kDa protein^5^ involved in various cellular mechanisms related to motor neuron development, including endocytosis,^6^ pinocytosis,^7^ endosomal dynamics,^8^ the lysosomal/autophagy pathway,^9^ and the stress-activated mitochondrial response.^10^ All those functions are important for the development and maintenance of the central nervous system, and dysfunctional ALSIN variants are linked to deficits in upper motor neurons.^11^

From a molecular point of view, ALSIN includes 4 domains (Figure 1A): the chromosome condensation 1 (RCC1)-like domain (RLD), the Dbl/Pleckstrin Homology domain (DH/PH), the Membrane Occupation and Recognition Nexus repeats (MORN), and the Vacuolar Protein Sorting 9 domain (VPS9). RLD has been implicated in membrane localization,^12^ while the DH/PH and VPS9 have been identified as respective guanosine exchange sites for the Ras-related C3 botulinum toxin substrate 1 (RAC1) GTPase and the Ras-related protein Rab-5A (Rab5), respectively^13^ (Figure 1A).

**Figure 1:**
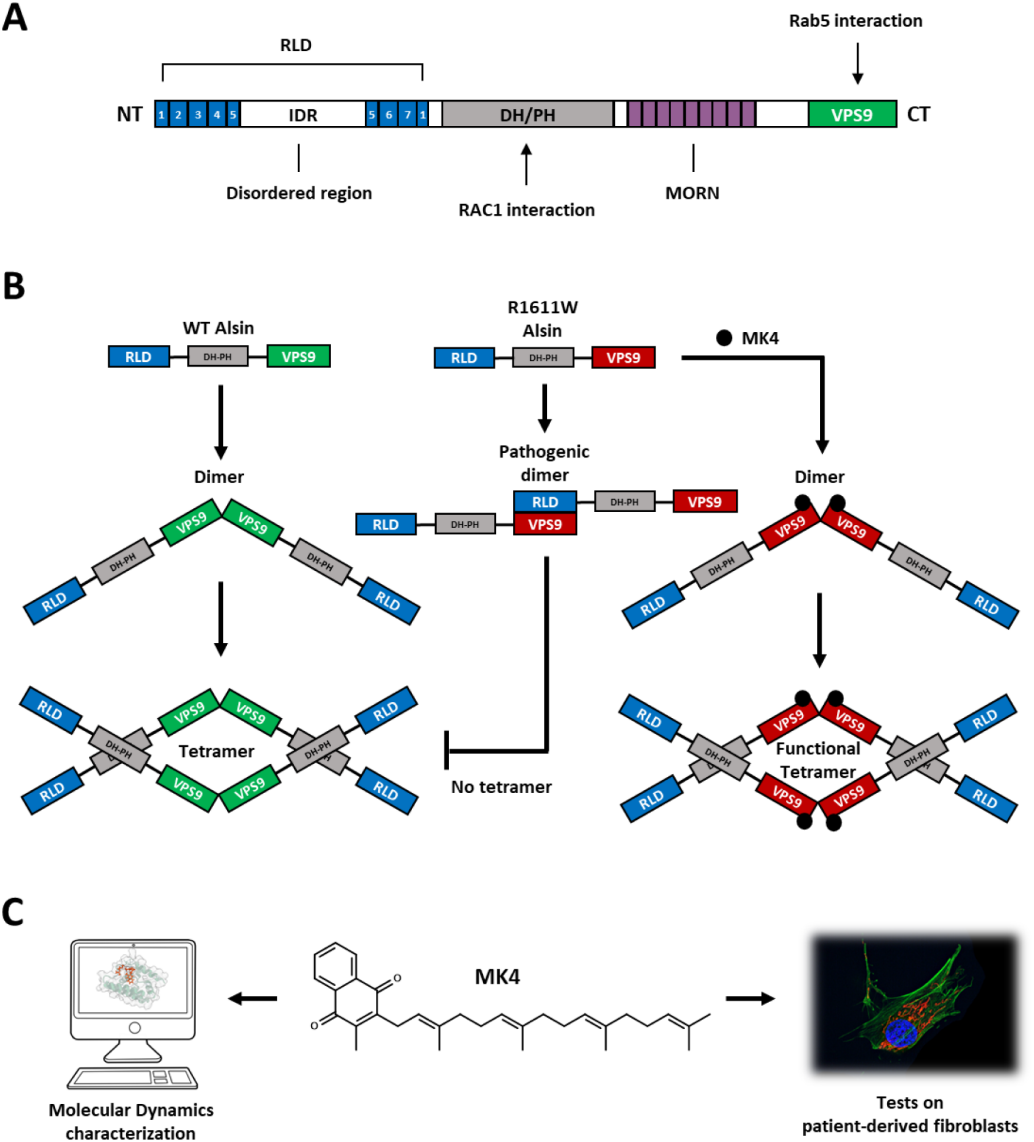
Alsin domain organization, tetramer assembly, and MK4 therapeutic strategy. (A) Schematic representation of ALSIN domains and known interactors. (B) Proposed model for tetramer assembly and pathogenic mechanism of R1611W mutant ALSIN corrected by MK4. (C) Brief outline of this work: computational and *in vitro* characterization of MK4’s effect.

The active form of ALSIN is a homotetramer, first assembled by dimerization through the VPS9 domains and subsequently by the interaction of two dimers through their DH/PH domains (Figure 1B, left).^14^ Of note, our previous work exploited protein modeling to reconstruct single domains and the 3D structure of ALSIN tetramers, despite the absence of experimental 3D models.^15^ This helped rationalize part of published biochemical experiments showing that different mutations, mainly involving the VPS9 domain, can affect tetramerization, leading to the accumulation of altered oligomeric states,^14,16^ impaired cellular localization,^12^ and interaction with other proteins.^6,17^

One mutation (c.4831C>T), leading to the R1611W missense variant on the VPS9 domain, was discovered in an Italian IAHSP patient (AO) in compound heterozygosis with c.4368delG, p.K1457Sfs*14, leading to a truncated form. We focused on the missense variant and discovered that the mutant protein forms abnormal dimers involving the mutant residue, impeding correct tetramerization (Figure 1B, right).^18^ To tackle this mutation, we applied an *in silico* drug repurposing strategy, identifying Menatetrenone (MK4, Figure 1C). MK4 is predicted to bind the mutant residue, mask its side chain, and rescue the correct formation of tetramers (Figure 1B).^18^ MK4 is approved as a drug in Japan for the treatment of bone density disorders,^19^ is suitable for administration in infants,^20^ and shows low toxicity.^21^ Given its safety, MK4 was approved in a compassionate use regimen for AO.^18^

Despite its potential relevance, the discovery of MK4 as a therapeutic strategy still requires further investigation to strengthen its reliability. Key aspects remain unresolved, including the ability of the R1611W mutant form of ALSIN to bind small molecules, the binding mechanism of MK4, and the validation of its efficacy. In conventional drug discovery pipelines, these questions are typically addressed through a combination of biophysical *in vitro* and *in vivo* studies. However, for rare diseases such as IAHSP, access to the chemical matter of the protein involved is often not feasible due to limited resources and technical limitations. Indeed, some of us observed technical challenges in expressing and stabilizing the full-length ALSIN protein (data not shown); thus, conventional biophysical approaches to directly assess MK4 binding are currently prevented. Moreover, mouse models carrying AO’s mutations are neither commercially available nor likely to recapitulate the human IAHSP phenotype, as even complete *ALS2* knockout models failed to reproduce key disease features.^22^ For these reasons, this study aims to overcome the lack of experimental accessibility by filling the aforementioned gaps with a combination of computational and cellular strategies.

In the first part of this work, we employed Molecular Dynamics (MD), a computational technique simulating the evolution of a chemical system over time (often referred to as molecular microscope^23^) to thoroughly investigate the behavior of the R1611W mutant VPS9 domain. First, we focused on assessing the presence of binding sites suited to accommodate small molecules. Then, we characterized the types of interactions established between MK4 and VPS9.

In the second part of this study, we conducted *in vitro* cellular assays. Patient-derived fibroblasts are our chosen experimental model, as they retain the patient’s genetic background, can be easily manipulated *in vitro*, and are often suited to study common cell mechanisms of several rare diseases.^24,25^ First, we assessed the expression of ALSIN to infer upon the suitability of fibroblasts as a cell model. Then, we measured oxidative stress levels, a hallmark of several neurodegenerative diseases, often associated with mitochondrial dysfunctions.^24,26,27^ Finally, we developed a dedicated protocol to characterize mitochondrial morphology in fibroblasts and to identify a cell marker allowing us to monitor the cellular effect of MK4.

Overall, this study demonstrates that *in silico* techniques can be used to address experimental gaps and be combined with *in vitro* assays to support the repurposing of small molecules for the treatment of ultrarare HSPs. This approach is expected to be relevant for developing new preclinical alternative drug discovery programs suited to monogenic rare diseases.^2^

## Results and Discussion

### Dynamic Behavior of ALSIN VPS9 Domain and MK4/VPS9 Complex

As explained in the Introduction, MK4 is capable of restoring tetramerization of the R1611W mutant ALSIN.^18^ We have identified MK4 with molecular docking experiments performed on a model of the mutation site, i.e., the C-terminal VPS9 domain.^18^ However, the docking approach employed did not consider the conformational flexibility of the target protein.^28^ To explicitly account for this limitation, and in the absence of tractable biophysical assays, we employed Molecular Dynamics (MD) simulations^29,31^ as an orthogonal computational tool to refine and stress-test our docking-based binding hypothesis. MD serves here to predict features of the cavity around residue 1611, support selective binding, and predict major MK4 interactions. These analyses provide a necessary consistency check, verifying that the structural conditions required for the proposed mechanism are robust.

#### Stability of Mutant VPS9

We considered the previously employed mutant and healthy control VPS9 models^18^ and subjected them to MD runs (see Methods and Figure S1). We observed an overall reduction of Root Mean Square Fluctuation (RMSF, see Figure S1B), in agreement with the previously reported indications of reduced flexibility by the R1611W variant.^18^ Moreover, clustering MD coordinates based on their Root Mean Square Deviation (RMSD) reveals a reduced conformational space by the mutant, with three main states in the mutant model as opposed to five in the control counterpart (Figure S1C–E), further supporting that R1611W stabilizes the VPS9 domain. This aspect is relevant since stable 3D protein conformations favor the interaction with ligands and facilitate their structure-based discovery,^2^ as for MK4.^18^

#### Investigation of the Binding Cavity

We previously suggested the presence of a binding pocket (or cavity) around the mutant residue by investigating a static VPS9 model, supporting the binding of MK4.^18^ A pocket is an almost necessary condition for a small molecule to bind; thus, we decided to test to what extent such a site is conserved during an MD simulation. To this aim, we employed MDpocket, a tool specifically tailored to identify binding pockets in MD trajectories and track their properties.^32^ MDpocket confirmed the presence of a binding pocket surrounding mutant tryptophan at position 1611 along the whole trajectory. Specifically, two subpockets were identified, as depicted in orange in Figure 2A. Of note, the residues forming the identified subpockets correspond to contact residues identified by the docking experiments that led to the identification of MK4 ^18^ (Table S1). Another relevant aspect is whether this binding pocket is present in the mutant VPS9 domain. Measurement of the empty volumes with MDpocket highlighted that the mutant cavity is significantly larger than the control counterpart along the whole MD trajectory (Figure 2B, Figure S2A,B). This supports binding selectivity toward R1611W mutant ALSIN.

**Figure 2:**
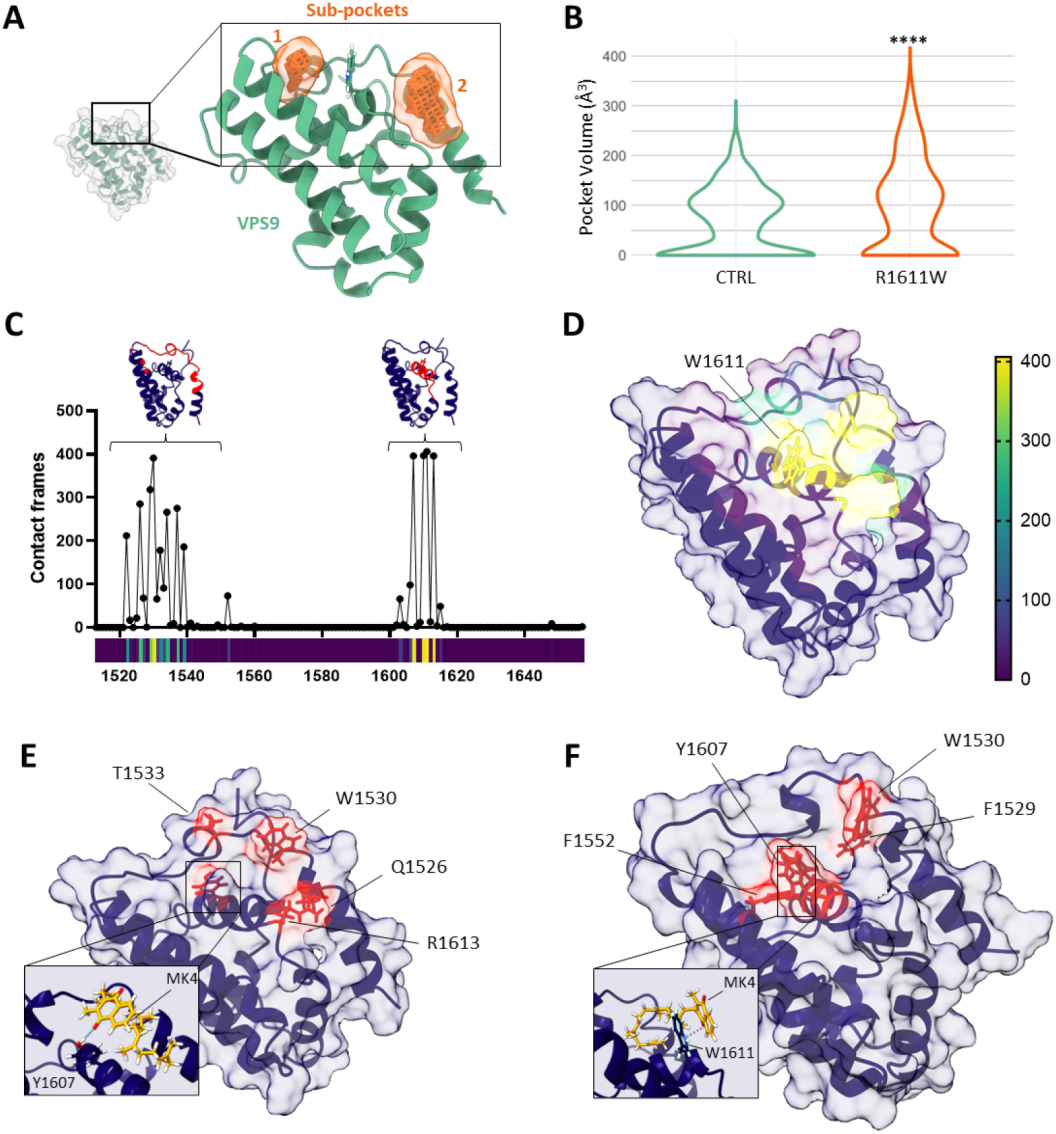
Molecular Dynamics analysis of the ALSIN VPS9 domain. (A) Analysis of MD trajectories of the VPS9 mutant model with MDpocket highlights two subpockets (in orange). (B) Pocket volume along MD trajectories of mutant and control VPS9 models. *p*-value from Wilcoxon’s *t*-test <0.0001. (C) Per-residue count of contacts between mutant VPS9 and MK4 in the MD simulation. (D) Projection of MK4’s contact frequency on the molecular surface of mutant VPS9 in the MD simulation. (E) Residues involved in hydrogen bond formation with MK4 in the MD simulation. (F) Residues involved in the formation of π-interactions with MK4 in the MD simulation.

#### Dynamic Interactions of MK4 with Mutant VPS9

In the absence of experimental binding data, the dissociation constant for the MK4/VPS9 complex was estimated with the neural network model KDEEP.^33^ The predicted midnanomolar range, in line with several small molecule drugs,^34^ provides supportive evidence for favorable interaction. To achieve a thorough description of how the binding occurs, we performed MD simulations starting from the initial mutant VPS9-MK4 docking complex (see Methods and Figure S3). When monitoring the distance between the center of mass of the ligand and pocket residues (see Supporting Information), it is suggested that MK4 overall interacts with VPS9 during most of the MD trajectory (ca. 65% of the sampled MD frames) by establishing an apparent equilibrium where it detaches and reinteracts with the receptor several times (data not shown). This behavior is coherent with what was previously described for some known drug-receptor complexes.^31^

Monitoring the most frequently involved contact amino acids (see Supporting Information) unraveled two main hotspots involving the amino acids in the 1520–1540 and 1600–1618 positions, with W1611 being the most contacted residue (Figure 2C, Table S2). Those two regions involve amino acids from the first amino terminal alpha helices interspaced by a loop (1520–1540) and by the α-helix containing W1611 (Figure 2C, highlighted in red). A visual projection over the molecular surface of the VPS9 domain model, colored by contact frequency, highlights an interaction patch surrounding mutant tryptophan (Figure 2D).

Next, we studied the type of interactions that MK4 establishes with mutant ALSIN. The molecular structure of MK4 (Figure 1C) suggests the ability to form hydrogen bonds (HBs) with the two oxygen atoms as potential hydrogen bond acceptors. To characterize the role of HBs, we performed an analysis with UCSF Chimera^35^ (see Methods). The residues involved in the HB events are T1533, W1530, Q1526, Y1607, and R1613, all in spatial proximity to W1611 (Figure 2E). However, out of the 1000 MD simulation frames, an HB event was recorded in only 17 frames, suggesting that this type of interaction is not the primary driving force of MK4 binding to mutant ALSIN. Being MK4 composed of an aromatic naphthoquinone moiety (Figure 1), we interrogated whether π-interactions play a role. To this end, we employed VMD^36^ (see Supplementary Methods) and sought for π-contacts involving residues with aromatic side chains. Unlike for HBs, we recorded a higher share of MD frames (121) with at least one π-interaction involving MK4. The π-contact residues are F1529, W1530, F1552, Y1607, and W1611 (Figure 2F), with the latter involving most contacts. Furthermore, we hypothesized that a strong contribution to MK4 interaction with mutant VPS9 comes from other types of interactions (i.e., hydrophobic contacts) since π-contacts and HBs are detected in less than one-third of the overall contact frames.

Overall, these analyses are consistent with the docking experiments that led to the identification of MK4 ^18^ and provide insights into its binding mode. While not replacing experimental binding measurements, these computational findings establish structural plausibility and identify testable features, such as mutant selectivity, framing our subsequent cellular validation strategy.

### Patient-Derived *In Vitro* Models: Characterization of IAHSP-Related Alterations

#### Establishment of a Patient-Derived Fibroblast Cell Line

We previously supported the discovery of MK4 with experiments demonstrating partial recovery of ALSIN tetramers.^18^ To test whether this is enough to recover ALSIN function at the cellular level, we here focused on patient-derived skin fibroblasts. Analysis of the publicly available database Human Protein Atlas^37^ shows that *ALS2* is expressed in this cell type, although at a lower level than in the central nervous system (Figure S4). We obtained consent from the family of an Italian IAHSP patient (AO) to perform a skin punch biopsy. The sampling is not excessively invasive, and it allowed us to obtain sufficient material to establish a fibroblast cell line after tissue disaggregation, resuspension, and plating (see Methods and Figure S5A).

To obtain a first prediction about the *ALS2* mRNA levels in the fibroblast line, we first ran a prediction with the NMDEsc Predictor^38^ suggesting that the mRNA from the K1457* allele (unlike R1611W) is not stable and undergoes Nonsense-Mediated RNA Decay (NMD). Specifically, the NMD border is predicted at base pair 4885, corresponding to residue 1628 (Figure S5B). For these reasons, we expect that AO expresses only the R1611W ALSIN variant.

#### ALSIN Expression in IAHSP Fibroblasts

We extracted total RNA from fibroblasts from the IAHSP patient AO and three healthy individuals and performed real-time quantitative PCR (RT-qPCR) with two separate oligo pairs. Oligo pair #1 is complementary to a region present in both alleles, while oligo pair #2 is specific to a region downstream of the missense mutation (Figure 3A, top). Data obtained with both primer couples showed that IAHSP fibroblasts express about 50% less *ALS2* mRNA than control cells (Figure 3A, bottom), suggesting that only the allele carrying the missense mutation is translated, while the other allele undergoes NMD. To assess ALSIN protein levels, we performed Western blot analysis on cell lysates from control and IAHSP fibroblasts and found an almost complete absence of ALSIN protein in IAHSP samples. Indeed, a faint signal corresponding to full-length ALSIN was only evident with a very long exposure time (Figure 3B). Immunocytochemistry experiments performed on fixed cells confirmed the trend but proved to be more sensitive: while control fibroblasts show a strong punctate intracellular pattern for endogenous ALSIN, as reported in literature,^7,14^ IAHSP cells showed a weaker, though detectable, signal (Figure 3C).

**Figure 3:**
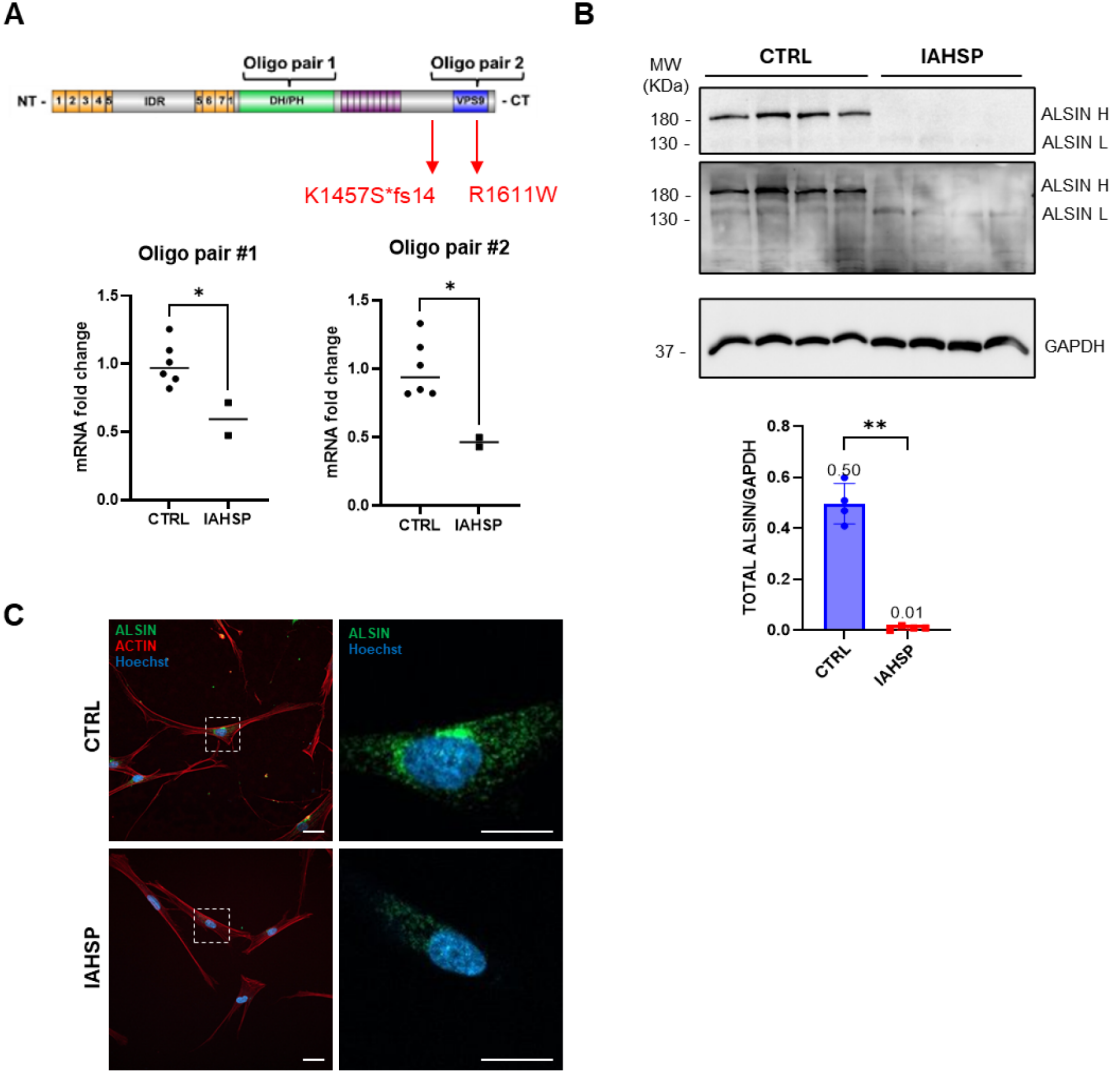
ALSIN expression in IAHSP fibroblasts. (A) Quantification of ALSIN mRNA levels by RT-qPCR. Top: schematics of the ALSIN sequence, indicating the regions spanned by the two oligo pairs. Bottom: RT-qPCR experiments comparing ALSIN mRNA levels from IAHSP and control fibroblasts. Data were normalized first to the housekeeping GAPDH gene and then to the control sample, set to 1. Unpaired Student’s *t*-test, *p < 0.05; *n* = 6 healthy subjects, *n* = 2 IAHSP. (B) Quantification of ALSIN protein levels by Western blotting analysis. Top: immunoblot of protein lysates from CTRL and IAHSP fibroblasts. Low and high exposure times are shown for ALSIN; anti-GAPDH antibody is used as a loading control. Data were normalized first to the intensity of the housekeeping GAPDH protein and then to the control sample, set to 1. Unpaired Student’s *t*-test, *p < 0.01; *n* = 4 biological replicates. (C) Representative confocal microscopy images of CTRL/IAHSP fibroblasts stained with anti-ALSIN antibodies (green channel), fluorescently labeled phalloidin to label actin cytoskeleton (red channel), and Hoechst to stain cell nuclei (blue channel). The high-magnification images show reduced ALSIN staining in IAHSP cells. Scale bars: 10 μm.

#### Oxidative Stress in IAHSP Fibroblasts

Neurodegenerative diseases are often characterized by increased oxidative stress,^39^ a feature also reported in HSPs.^40,41^ To test whether IAHSP fibroblasts experience oxidative stress, we measured cellular Reactive Oxygen Species (ROS) levels using a plate-based assay (see Methods).

Monitoring ROS production of IAHSP and control fibroblasts over 2 h revealed increased ROS levels in IAHSP cells (Figure 4A, blue and yellow curves). Analogous to what was performed by other authors,^10^ we also introduced exogenous oxidative stress by treating the cells with increasing concentrations of H_2_O_2_ and observed higher ROS production in IAHSP cells treated with 8–16 mM H_2_O_2_ compared to control cells, quantified by estimating “Area Under the Curve” (AUC) values (Figure 4B). These data suggest that IAHSP cells are characterized by an impaired capability to cope with oxidative stress.

**Figure 4:**
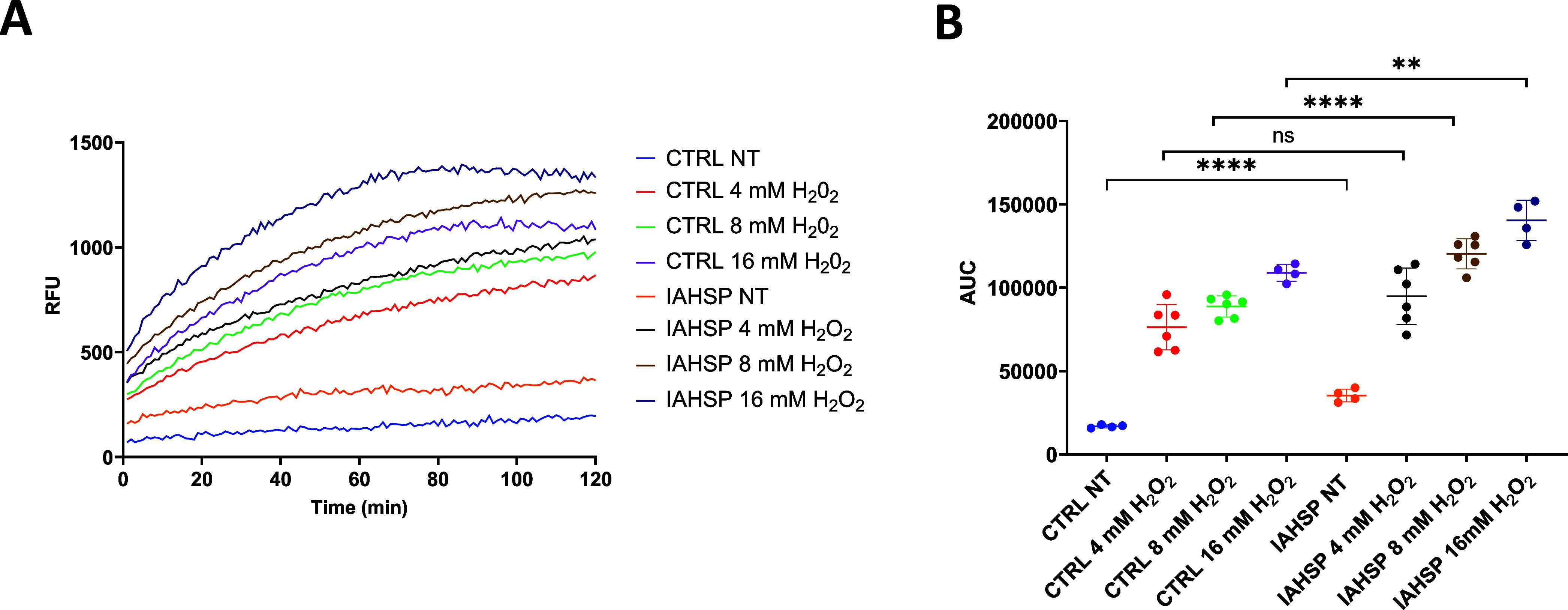
IAHSP fibroblasts show increased oxidative stress and increased susceptibility to oxidative stimuli. (A) Kinetic curves reporting the increase of fluorescent signal (expressed in Relative Fluorescence Units, RFU) quantifying ROS levels under basal conditions (NT: not treated) and upon exposure to increasing H_2_O_2_ concentrations, as indicated, in CTRL and IAHSP cells. (B) Area Under the Curve from data reported in (A). Unpaired *t*-test; **p < 0.01, ****p < 0.001, *n* = 4–6 biological replicates. NT = not treated.

#### Mitochondrial Morphology in IAHSP Fibroblasts

Oxidative stress is often associated with mitochondrial defects,^42^ and mitochondria themselves play pivotal roles in neurodevelopment.^39^ Moreover, ALSIN-dependent Rab5 localization to mitochondria correlates with their stress responses.^10^ Thus, to identify a potential cellular marker of pathology, we examined the mitochondrial morphology in IAHSP and control fibroblasts.

We set up a protocol to stain mitochondria, acquire adequate numbers of super-resolution microscopy Z-stack images, automatically reconstruct mitochondrial networks, and measure mitochondrial descriptors (see Methods and Figure S6A). Specifically, the measured descriptors were obtained from the ImageJ Mitochondria Analyzer plugin,^43^ and they allow us to characterize both pure morphological (e.g., size, shape) and network (e.g., branching, thickness) parameters (more details in Supporting Information). In line with our previous experiments, we assessed mitochondrial morphology both under steady and stressed conditions by treating the cells with H_2_O_2_.^44^

Mitochondria Analyzer offers the possibility to reconstruct mitochondrial networks both in 2D and 3D. The former method is computationally more accessible; thus, we performed an initial characterization of 2D images, staining mitochondria with the MitoTracker dye.^43^ Despite several optimization attempts, this setup resulted in several artifacts, mainly related to the apparent erroneous identification of background signals (Figure S6B). Even though less prone to artifacts, 3D reconstructions of the same images were still not optimal (data not shown). To overcome the limitations, we employed the mitoDendra2 plasmid.^45^ This system drives the expression of a mitochondrion-localized version of the photoconvertible fluorescent protein Dendra2, thus allowing visualization of mitochondria in the green/red fluorescent channel (Figure 5A).^45^ We cultured IAHSP and control fibroblasts with or without H_2_O_2_ ^38^ and acquired super-resolution images. 3D mitochondrial networks were reconstructed, and 16 morphological and network parameters (Table S3) were determined with the Mitochondria Analyzer plugin (see Methods).^43^

**Figure 5:**
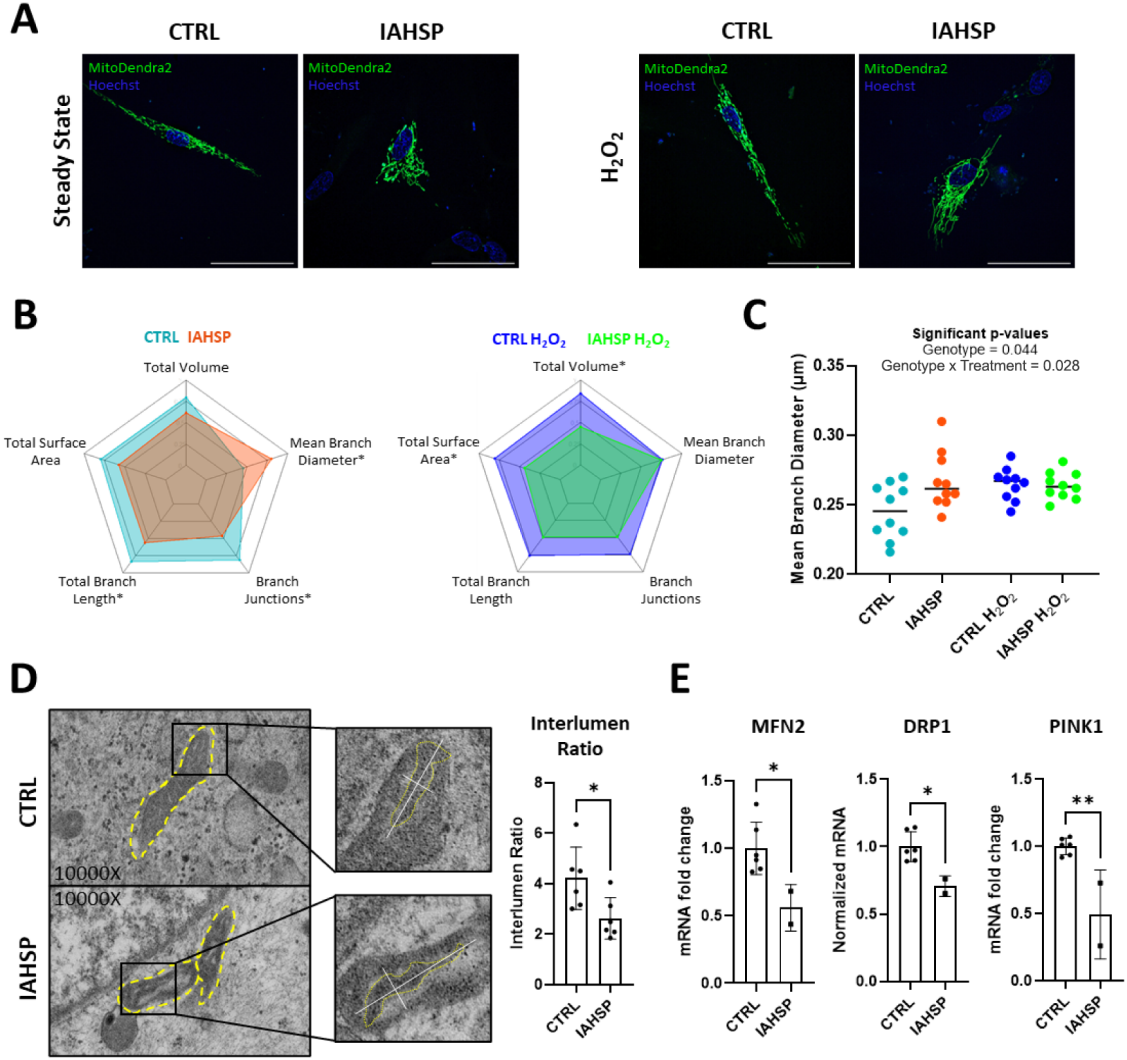
Mitochondrial deficits and oxidative stress in IAHSP fibroblasts. (A) Representative images of control and IAHSP fibroblasts, mitochondria in green (MitoDendra2), nuclei in blue. Scale bars: 50 μm. (B) Average values of selected mitochondrial parameters; statistically significant differences are marked by *p < 0.05. Statistical analyses performed with Wilcoxon’s *t*-test on 10 independent observations. (C) Multiple comparisons of Mean Branch Diameter from control and IAHSP fibroblasts with and without H_2_O_2_ treatment. Statistical test: two-way ANOVA followed by Tukey’s multiple comparisons test; *p*-values as indicated. Independent observations: *n* = 10 per group. (D) Representative TEM images of control and IAHSP fibroblasts and interlumen ratio quantification; yellow dashed lines highlight mitochondria. Statistical analyses performed with Wilcoxon’s *t*-test, *p < 0.05, *n* = 6. (E) RT-qPCR quantification of MFN2, DRP1, and PINK1. Statistical analyses performed with Wilcoxon’s *t*-test, *p < 0.05.

Selection of five statistically significant parameters (Figures S7 and S8) and visualization of their average values reveals differences in branching and mitochondria content. Specifically, we report lower Total Branch Length, fewer Branch Junctions, and increased Mean Branch Diameter (MBD) in IAHSP fibroblasts relative to the healthy control (Figure 5B, left). Additionally, trends indicating reduced mitochondrial content (total mitochondrial volume and surface area) were observed in steady-state IAHSP fibroblasts compared to healthy controls (Figure 5B, left). These data suggest that IAHSP mitochondria are overall shorter, less networked, and thicker, all features connected with mitochondrial suffering.^39^ Most of these differences are exacerbated by the application of exogenous oxidative stress with H_2_O_2_ treatment (Figure 5B, right), and a statistical analysis considering all four conditions together consistently highlights a genotype effect (Figure S9).

An exception is represented by the parameter MBD, which is illustrated in Figure 5C. Statistical comparison reveals that under steady-state conditions, IAHSP fibroblasts have higher MBD than control cells (two-way ANOVA followed by Wilcoxon’s *t*-test, *p*-value = 0.028). However, under H_2_O_2_ treatment, this difference is no longer present. This suggests that mitochondria in IAHSP fibroblasts are already as thick as those in stressed control cells, an alteration previously linked to poor mitochondrial functionality.^39^ We hypothesized that this analysis identified a cell marker to monitor the effectiveness of candidate drugs to restore ALSIN’s function.

To delve into mitochondrial morphology, we analyzed control and IAHSP fibroblasts with Transmission Electron Microscopy imaging (TEM, Figure 5D). Measurement of the Feret Diameter per mitochondrion (a measure of size) and the Aspect Ratio (a measure of shape) suggests thicker mitochondria with altered shapes in IAHSP fibroblasts (Figure S10A), in agreement with the MBD data illustrated in Figure 5B,C. Moreover, we analyzed the intramitochondrial morphology and determined interlumen ratio values (ratio between cristae and empty area). The area occupied by mitochondrial cristae is proportional to the efficiency of these organelles,^46^ and we measured reduced values for IAHSP fibroblasts compared to control (Figure 5D). Furthermore, we observed that cristae of IAHSP mitochondria are longer and thinner than those in control cells (Figure S10B). Overall, the TEM analysis depicts a situation where IAHSP mitochondria are enlarged and display longer and thinner cristae. This is often an indicator of mitochondrial suffering.^47^

The previous data overall indicate that mitochondria from IAHSP fibroblasts behave differently from controls, strongly suggesting aberrant function and lower mitochondrial quality. Following a previous investigation highlighting fission and fusion impairment in *ALS2* knockout models,^10^ we verified whether this also occurs in IAHSP fibroblasts. We employed RT-qPCR to assay the expression of markers of mitochondrial fusion (MFN2 ^48^), fission (DRP1 ^48^), and mitophagy (PINK1 ^49^), the cellular disposal mechanism responsible for maintaining the mitochondrial pool. We observed a 50–60% mRNA reduction in IAHSP fibroblasts for all three markers, suggesting impairment of mitochondrial dynamics and lower mitochondrial quality control (Figure 5E).

Overall, the data indicate a specific phenotype linked to mitochondria, which can be employed as a cellular marker to monitor the efficacy of treatments in IAHSP fibroblasts.

#### MK4 Administration: Effect on Mitochondria and ALSIN Levels

Aiming to test whether MK4 can revert the previously described mitochondrial phenotype, we treated control/IAHSP fibroblasts with 5 μM MK4 for 72 h, replicating the experimental setting shown in Figure 5A. We then acquired super-resolution images of MitoDendra2-transfected cells (Figure 6A), performed 3D reconstruction, and determined the Mean Branch Diameter under the four experimental conditions. Our analysis revealed the rescue of enlarged mitochondria in IAHSP cells, suggesting the selective activity of MK4 (Figure 6B).

**Figure 6:**
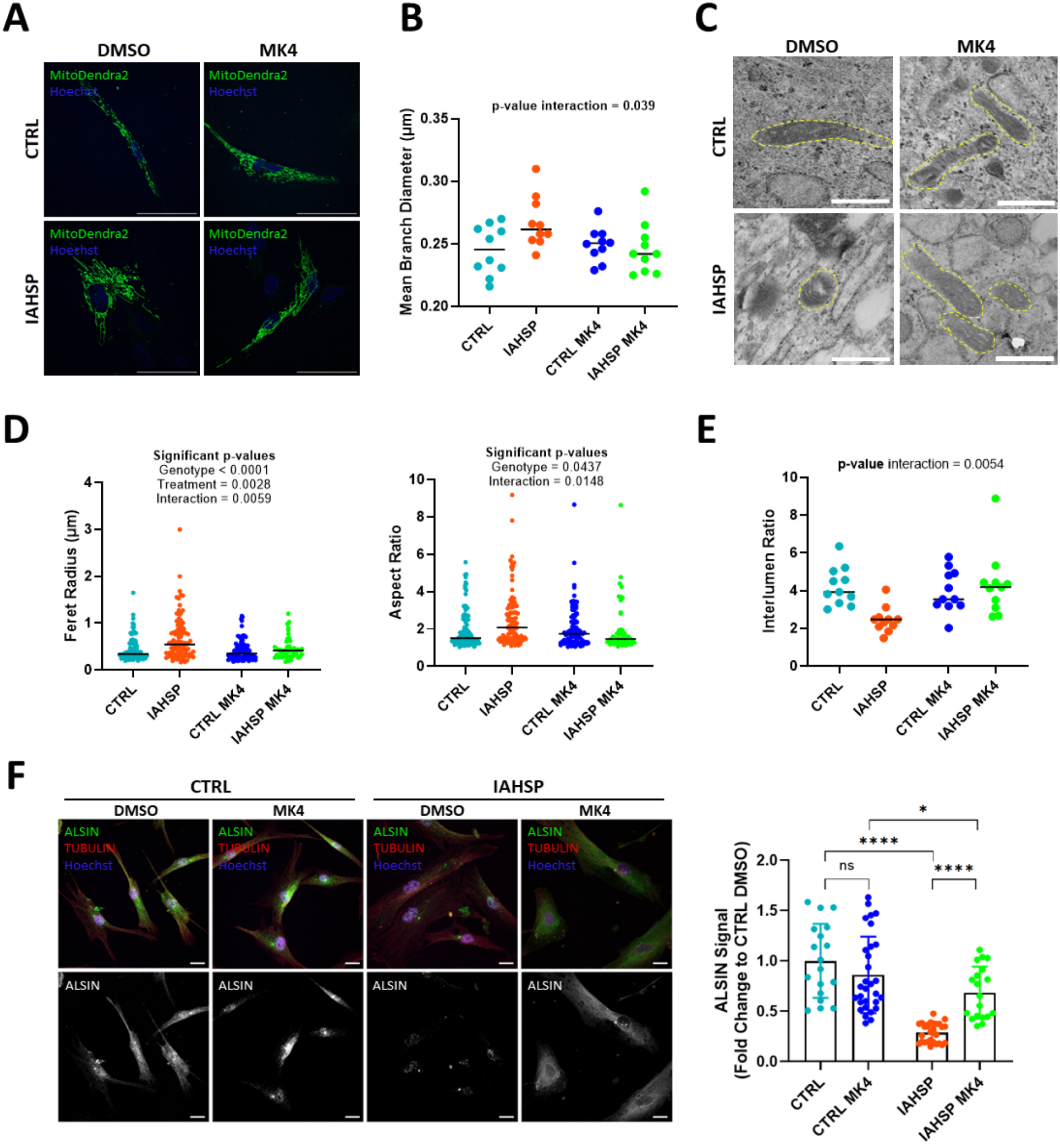
MK4 treatment rescues mitochondrial morphology and ALSIN levels. (A) Representative images of control and IAHSP fibroblasts treated with 5 μM MK4; mitochondria in green (MitoDendra2), nuclei in blue. Scale bars: 50 μm. (B) Analysis of Mean Branch Diameter from control and IAHSP fibroblasts with and without MK4 treatment; independent observations: *n* = 10 per group. (C) Representative TEM images of control and IAHSP fibroblasts; yellow dashed lines highlight mitochondria, scale bars: 1μm. (D) Multiple comparisons of Feret Radius and Aspect Ratio values from TEM images; independent observations: *n* = 100 per group. (E) Multiple comparisons of Interlumen Ratio values from TEM images; independent observations: *n* = 10 per group. Statistical analyses performed with two-way ANOVA followed by Tukey’s multiple comparisons test; *p*-values are reported on the graphs. (F) Left: representative confocal microscopy images of CTRL and IAHSP fibroblasts treated with 10 μM MK4 in 0.1% DMSO or 0.1% DMSO and stained with anti-ALSIN (green channel), antitubulin (red channel, cytoskeleton), and Hoechst to stain cell nuclei (blue channel). Scale bars: 10 μm. Right: statistical analysis performed with two-way ANOVA followed by Tukey’s multiple comparisons test; *p*-values from multiple comparisons are indicated by * (*p*-value < 0.05: *, *p*-value < 0.001: ****); *n* = 18–30 cells.

To further confirm the effect of MK4 on the mitochondrial morphology, we performed TEM imaging (Figure 6C) and determined mitochondrial morphological parameters. Analysis of the Feret Diameter shows an increased size for IAHSP mitochondria, which is reverted upon MK4 treatment (Figure 6D, left). Similar results are found when determining the Aspect Ratio, which is higher for IAHSP mitochondria and selectively rescued by treatment with MK4 (Figure 6D, right). Taken together, these data support the notion that IAHSP mitochondria are thicker and that MK4 can correct their morphology.

To also investigate the effect of MK4 on intramitochondrial morphology, we determined the interlumen ratio in TEM images, as in Figure 5D. Also in this case, treatment with MK4 brings the interlumen ratio values to control levels, supporting rescue of mitochondrial function (Figure 6E).

To assess the impact of MK4 on ALSIN expression, we utilized immunocytochemistry. The representative images in Figure 6F show a partial recovery of ALSIN signal intensity in treated IAHSP fibroblasts (Figure 6F left, green channel and gray panels). This was confirmed by quantification (Figure 6F, right), suggesting that MK4 contributes to the stabilization of ALSIN, partially restoring its levels in IAHSP fibroblasts.

Overall, the data support that MK4 treatment improves specific aspects of both mitochondrial morphology and functionality in IAHSP cells by directly acting on mutant ALSIN.

## Conclusion

A preclinical approach to single-patient cases harboring rare mutations poses several challenges. First, no standard protocols are available, meaning that *ad hoc* studies should be set to identify a cell marker and test for drug candidates. Second, published experimental data is limited, as are time and resources for a broad experimental investigation.^2^ Third, the experimental feasibility may be constrained by technical limitations or by the lack of appropriate *in vivo* models. For these reasons, in this work, we employed a mix of complementary *in silico* and cell-based techniques supporting our previous findings about the beneficial effects of MK4 treatment for IAHSP-causing ALSIN mutation R1611W.

We started by analyzing a previously published 3D protein model of the VPS9 ALSIN domain containing the R1611W missense mutation^18^ by means of MD simulations. We characterized the binding pocket centered around the mutant residue, highlighting the presence of two subpockets, which remain open and ligand-available just in the pathogenic variant, supporting the selectivity of MK4. Moreover, we characterized the VPS9 residues contacted by MK4 and the type of established interactions, identifying residue 1611 as the most contacted and supporting MK4 activity in shielding this mutant residue. These results, together with the prediction of a theoretical dissociation constant, support the binding of MK4 and the idea that experiments at commonly used concentrations (e.g., low-micromolar range) could be successful for testing its action on cells.

We established an *in vitro* cultured, patient-derived skin fibroblast line and demonstrated that ALSIN is expressed in this cell type. Furthermore, we provided evidence suggesting that the missense variant is expressed in IAHSP fibroblasts, albeit at minimal levels, partially confirming predictions and supporting the use of MK4 as a therapeutic strategy.^18^ Fibroblasts offer several advantages when testing the efficacy of a drug candidate,^2^ as they are easy to culture and reach confluency within a few days; thus, experiments can be fast-paced. Moreover, being patient-derived, fibroblasts share the same genetic background as the patient, thus reducing variability caused by interindividual differences.^26^ However, fibroblasts are significantly different from neurons; thus, in future experiments, it will be essential to verify that similar cellular mechanisms are reflected in this cell system.

We report a state of increased oxidative stress in IAHSP fibroblasts, consistent with previous reports in *ALS2* knockout models^50^ and other neurodegenerative^39^ and motor neuron diseases.^51^ In parallel, we observed alterations in mitochondrial morphology along with dysregulated expression of genes involved in mitochondrial maintenance. Together, these findings support prior evidence linking ALSIN to the mitochondrial stress response,^10^ suggest a condition of mitochondrial distress,^52,53^ and support that mitochondria-related readouts can be employed to monitor the effect of drugs in this context.

To characterize mitochondrial architecture in detail, we developed a dedicated 3D imaging and analysis protocol, enabling high-resolution reconstruction of mitochondrial networks. Using this approach, we identified an increased Mean Branch Diameter as a distinctive morphological feature of IAHSP fibroblasts. While a full mechanistic dissection is beyond the scope of this study, our goal is to establish a robust cellular marker suitable for monitoring drug-induced effects *in vitro*. We therefore propose the Mean Branch Diameter (MBD) as a candidate marker.

Treatment of IAHSP fibroblasts with MK4 led to a measurable improvement in MBD and ALSIN levels, indicating an effect specific to the IAHSP condition and supporting the beneficial impact of the compound. MK4 is a molecule with reported roles in various cellular processes, including mitochondrial pool maintenance^54,57^ and antioxidant defense.^58^ Thus, we cannot exclude the possibility that these pathways also contribute to the observed phenotypic rescue. However, here we show a direct effect on ALSIN levels, while antioxidant or electron transport chain-modulating therapies have shown limited and not fully understood effects in similar neurodegenerative diseases.^59,62^ Taken together with the evidence collected here and the previously reported specific effect on mutant ALSIN tetramers,^18^ these observations support a fundamental role played by ALSIN-mediated mechanisms.

This study focuses on AO, a single patient; therefore, the issue of representativity of the identified cell marker over wider IAHSP cohorts remains. For this reason, we envision a next step undertaking joint collaborative efforts to build an IAHSP patient data set and establish a matching fibroblast biobank. This would ensure a wider coverage of different mutations, and the lean culture protocols of skin fibroblasts would provide an abundance of study material. If this were the case, high-throughput techniques could be routinely employed as performed for similar diseases.^25^ Moreover, systematic studies of mitochondria could help dissect all underlying mechanisms and identify further biomarkers. Finally, a broader drug repurposing campaign could be attempted, mirroring the *in silico* pipeline that identified MK4.^18^ This approach may lead to unique gains of information, improving drug repurposing protocols for rare diseases, such as IAHSP.

## Materials and Methods

### Molecular Modeling Procedures

#### VPS9 Modeling

The 3D model of the ALSIN VPS9 domain was obtained as previously reported.^18^ As described in the original publication,^18^ the ALSIN VPS9 region was modeled through a three-step strategy in which the VPS9 core domain (residues 1513–1649, containing the Rab5-GEF region), the helical bundle (HB, residues 1392–1512), and the intervening 20-residue linker were independently predicted. The ALSIN sequence (UniProt ID: Q96Q42) was retrieved from UniProt (accessed 9 March 2022), and domain boundaries were defined by sequence alignment with human Rabex5.^18^ The VPS9 core (residues 1513–1657) was modeled using Modeler (salilab.org/modeller) integrated in UCSF Chimera, with PDB 2OT3 and 1TXU as templates and default parameters. The HB domain was generated with the MOE homology model wizard (www.chemcomp.com, accessed 5 September 2022), employing PDB 2OT3 as a template, and aligned to the Rabex5 crystal structure (PDB 1TXU) to define the relative orientation of the VPS9 and HB models. The linker (residues 1493–1513, sequence ALDNDREEDIYWECVLRLNK) connecting the HB and VPS9 domains was modeled using the MOE linker modeler wizard, based on PDB 3LP8. Local and global minimizations were carried out with the AMBER 12:EHT force field, implicit solvation, and R-field parameters, according to the manufacturer’s recommendations. The final wild-type and R1611W mutant models encompassed residues 1392–1657.

#### Docking Procedures

The structure SMILES for MK4 was retrieved from the DrugBank website (https://go.drugbank.com, last accessed on 12.04.2021). The docking was performed with GOLD^63^ (www.ccdc.cam.ac.uk, accessed on 1 May 2021) version 2020.2.0. The cavity parameters were centered on W1611 in the previously described VPS9 3D model; point: 15.433, 6.723, 5.661; rest: default parameters in the wizard. The scoring function was the fitness based on ChemPLP, as previously reported.^18^

#### Molecular Dynamics Simulations

The starting structure for MD simulations encompasses the VPS9 core region (predicted to bind MK4 by docking experiments). The input preparation for the MD protocol was generated with CHARMM-GUI^64^ (https://www.charmm-gui.org, last accessed on 10.10.23) in explicit water solvent, with an ionic strength of 0.15 M, as previously described.^65^ The simulation was run on NAMD,^66^ vv2.14, CUDA-accelerated version (www.ks.uiuc.edu/Research/namd). Equilibration was carried out for 250 ps (NVT ensemble), and the total production (NVT ensemble) length was 100 ns. The simulations were run on a Linux workstation (OS: Ubuntu 22.04, CPU: Ryzen 5000, 3.50 GHz, RAM: 32GB DDR2, GPU: RTX A2000 12GB). The trajectories were initially aligned, the solvent molecules removed by employing VMD^36^ and analyzed with the Bio3D R package^67^ as proposed by the most recent pipeline from the Grant Lab (http://thegrantlab.org/, last accessed on 1 September 2023). Clustering of MD states was carried out based on RMSD by using the Python library ttclust^68^ with default settings.

#### Pocket and Contact Analysis

MDPocket^32^ was employed to search for binding cavities on control and mutant VPS9 models in two rounds. First, the default settings were run on the trajectory to identify the pockets. Then, a selection of the generated dummy atoms from the output pdb file “mdpout_freq_iso_0_5.pdb” was made upon visual inspection. These dummy atoms identify volumes that remain open in at least 50% of the analyzed MD frames. The selected dummy atoms were included in a second command run to extract descriptors and specific features of the identified pockets surrounding residue 1611.

Total and π-contacts were identified in VMD^36^ using the scripts provided in Supporting Information. Hydrogen bonds were identified using UCSF Chimera (vv 1.16) and iteration through the MD movie panel of the command “hbond”.

#### Affinity Prediction

The affinity prediction with the graph-based neural network model KDEEP^33^ was carried out on the best 10 docking poses submitted through the web interface (https://playmolecule.com/Kdeep, last accessed 12.12.23).

### *In Vitro* Assays

#### Skin-Derived Fibroblast Primary Cultures

A stock solution of Collagenase (C6885-Sigma) was prepared by dissolving 0.16 g in 10 mL of sterile Milli-Q water and stored at −20 °C. Dulbecco’s Modified Eagle’s Medium (DMEM, 11965092, Gibco) was supplemented with 10% Fetal Bovine Serum (FBS, A5256701, Gibco), Penicillin-Streptomycin (PenStrep, 15140122, Thermo Fisher), and Sodium Pyruvate (11360070, Thermo Fisher); this complete medium was stored at 4 °C. A collagenase working solution was freshly prepared by diluting the collagenase stock solution to 10% (v/v) in complete DMEM medium, followed by sterile filtration.

A skin biopsy, obtained via a skin punch, was collected in a 15 mL sterile tube containing DMEM, Pen-Strep, and Amphotericin B (Cat. 15290018, Gibco). The sample was then aseptically transferred to a Petri dish using a needle and scalpel, finely minced, and washed with Phosphate-Buffered Saline (PBS). The resulting tissue pellet was resuspended in the collagenase working solution and incubated for 24 h at 37 °C. Following incubation, the tissue was further disaggregated by pipetting and centrifuged, and the cell pellet was resuspended in fresh complete cell culture medium. Adherent cells were subsequently cultured in T-25 flasks containing DMEM supplemented with 10% FBS, sodium pyruvate, and Pen-Strep. Cultures were maintained at 37 °C in a 5% CO_2_ humidified incubator and passed up to a maximum of 15 times. Cells were dissociated for passaging or experiments using 0.25% Trypsin-EDTA supplemented with phenol red (25200056-Gibco).

#### Handling of MK4 Chemical Matter

MK4 (Sigma-Aldrich) was solubilized in a DMSO stock solution at 20 mM (far above what was expected), and the stock solution was directly diluted up to a maximum of 20 μM in cell culture media without precipitation.

#### Mitochondrial Staining

For the mitochondrial staining, 10000 cells were seeded on round glass coverslips in 24-well plates and allowed to adhere for 24 h. The next day, cells were treated by adding new full media with 5 μM MK4. For the MitoTracker experiments, after 72 h of treatment, the cell medium was removed and replaced with MitoTracker CMX Red (Thermo Fisher) working solution (1:500) in full media and incubated for 1 h. Then cells were fixed and stained with Phalloidin and Hoechst as described below.

The expression of the tagged construct in the MitoDendra2 plasmid (Addgene ID #55796) occurred via transfection by means of Lipofectamine 2000, as described elsewhere.^45^ Transfection was carried out on day 2, 24 h after plating (and before starting treatment with MK4).

#### Immunofluorescence Analysis

For fluorescence microscopy, 10000 cells were seeded on round coverslips (24-well plates), cultured, and fixed with PFA as described before.^69^ Coverslips were then washed, and cells were permeabilized with 0.1% v/v Triton-X100 (X100-SigmaAldrich) in PBS, incubating for 10′. Cells were washed, blocked in 5% BSA/0.1% Triton-X100, and then stained with Phalloidin AlexaFluor-647 (cytoskeleton staining, A22287) and anti-ALS2 antibody (EPR27469-29, Abcam ab316195) for 2 h at RT in blocking solution. Following washing, secondary antibody incubation was performed with AlexaFluor-488 (Thermo Fisher, A-11094) in washing buffer 0.1% Triton-X100 for 1 h at RT. Nuclei were stained with a Hoechst (33342-Invitrogen) 100 ng/mL PBS solution. Finally, after PBS washing, coverslips were mounted on microscopy slides using Fluoromount mounting media (F4680-Sigma). Images at 40× were acquired on an Elyra 7 microscope (Zeiss).

#### Gene Expression Analysis

RNA was extracted from 10^5^ cells plated in 6-well plates on the day after plating. After snap freezing at −80 °C, RNA was extracted with the TRIzol reagent (15596026-Invitrogen) coupled with chloroform (650498-Sigma), according to the manufacturer’s protocols. cDNA synthesis was carried out with the First Strand cDNA Synthesis Kit (K1612-Thermo Fisher), with 500 ng RNA as the template. For the rest, we followed the manufacturer’s protocols. RT-qPCR experiments were performed by means of the Master Mix Fast Start SYBR Green (FSSGMMRO-Sigma-Aldrich). Each reaction well contained a total volume of 20 μL, composed of 1 μL of template, 2 μL of primer solution (10 mM), 7 μL of nuclease-free water, and 10 μL of SYBR Green. The primers were designed with the NCBI primer design tool (https://www.ncbi.nlm.nih.gov/tools/primer-blast, last accessed 31.05.23) and were synthesized by Invitrogen. The sequences are the following: Oligo Pair 1 fw: TACAGTCCAGCTGTTGCAGG; Oligo Pair 1 rv: CCCATGAAGGAAGCTGCTCA; Oligo Pair 2 fw: GTGCTACGGGCCAGGATTAG; Oligo Pair 2 rv: TGCCTTCAAGGTGGTGAACA; PINK1 fw: CAAGAGAGGTCCCAAGCAAC; PINK1 rv: GGCAGCACATCAGGGTAGTC; MFN2 fw: TGCAGGTGTAAGGGACGATT; MFN2 rv: GAGGCTCTGCAAATGGGATG; DRP1 fw: CAAAGCAGTTTGCCTGTGGA; DRP1 rv: TCTTGGAGGACTATGGCAGC. Analysis of RT-qPCR experiments was carried out with the ΔΔCT method.

#### Western Blot Analysis

Primary fibroblasts were plated in T25 tissue culture flasks and harvested by trypsinization at 80% confluency. The cell pellet was then lysed in 1X RIPA lysis buffer and subjected to 4 sonication cycles with an amplitude of 10% for 10 s. Samples were spun to remove debris, and total protein from the supernatant was quantified with the Bradford assay (Bio-Rad, 5000006). 30 μg total proteins were resolved by 10% SDS-PAGE and transferred onto a nitrocellulose membrane (Amersham, GE10600001). After Ponceau S staining and washing, the membrane was blocked in 5% BSA/0.1% TBS-Tween20 for 1 h at RT. It was then incubated O/N at +4 °C with the primary antibodies anti-ALS2 (Abcam, ab316195) and anti-GAPDH (Cell Signaling, 2118S). After 3 × 5′ washing in 0.1% TBS-Tween20, the membrane was incubated with the appropriate HRP-conjugated secondary antibody for 1 h at RT and developed in a ChemiDoc apparatus (Bio-Rad) using ECL Star (Euroclone, EMP001005).

#### Reactive Oxygen Species Quantification

ROS titration was carried out with the cellular ROS assay kit from Abcam (catalog ab186027). In brief, 5.0 × 10^3^ cells were seeded in black, clear-bottom, 96-well plates (165305-Thermo Fisher) and incubated overnight at 37 °C with 5% CO_2_. On the day of the assay, the culture medium was removed, cells were rinsed in 1X PBS, and then assay buffer was added and incubated for 1 h at 37 °C with 5% CO_2_. ROS reading was performed following the manufacturer’s instructions with a FLUOstar Omega instrument from BMG LABTECH with excitation/emission wavelengths of 520/605 nm, respectively, in kinetic mode (one read per minute) in incubation at 37 °C for 2 h.

ROS acquisition was carried out in quadruplicate, and data analysis was performed by subtracting blank values at each time point in order to generate kinetic curves and calculate the Area Under the Curve (AUC) used to assess the changes between control and IAHSP condition.

#### TEM Imaging

Cells were cultured in T75 ventilated flasks until they had 90% confluence. On the day of sample preparation, cells were rinsed twice with 1X PBS and detached with 0.25% Trypsin-EDTA (1X) (Thermo Fisher, cat. 25200056). Cells were then centrifuged for 5′ at 1000 rpm to form a pellet, and the supernatant was removed and substituted with fixative solution 2.5% Glutaraldehyde in 200 mM Na-Cacodylate buffer at pH 7.3 for 45′. Fixative was removed, and pellets were washed overnight in 200 mM Na-Cacodylate buffer, followed by secondary fixation in 1% osmium tetroxide (OsO_4_). Samples were dehydrated in graded ethanol, transferred to a transitional fluid (propylene oxide), and then infiltrated and embedded in Agar 100 epoxy resin. Polymerization was at 60 °C for 48 h. Ultrathin sections (90 nm) were cut by using a Diatome diamond knife on a Leica Ultracut UC7 ultramicrotome (Leica Microsystems, Germany). Sections were picked up on Athene 300 mesh copper grids and stained with 70% alcoholic uranyl acetate and Reynold’s lead citrate for contrast.

Independent images from randomly selected control or patient fibroblasts were studied. Initially, fibroblasts from each culture were examined at ×1000 magnification. Representative cells showing particular ultrastructural features were recorded at higher magnifications for further assessment. Interpretation of TEM changes was made by comparing patient-derived samples with the control sample that was processed in parallel (sample preparation + acquisition). Imaging was performed on a Philips EM 208 Electron Microscope.

For the analysis of TEM images, each image was manually opened as a tiff file, and ROIs were drawn manually. Two types of selections were made: per mitochondrion and intramitochondrion (cristae/empty volume). Each ROI was separately saved, and geometric/morphological descriptors were calculated with the “Analyze-Measure” ImageJ tool.

#### Image Processing and Measurement of Mitochondrial Parameters

Super-resolution z-stack images were exported as czi files and opened as hyperstacks with ImageJ (vv 1.53t). For the bidimensional analysis, the channel of the mitochondrial staining was z-projected as the average intensity and converted to 8-bit images. ROIs containing single cells were manually drawn, and the background was removed. Then, the ImageJ plugin “Mitochondria Analyzer”^43^ was opened, and a 2D threshold was defined with default settings. Then, the 2D analysis with default settings was performed on thresholded images by choosing the “per mito basis” option. After looking at the data distribution (data not shown), the decision to average the data per cell was taken, and the reported data are per cell. Concerning the 3D analysis, a similar process was followed (ROI and cell selection), but the 3D batch analysis option was chosen (thresholding + analysis) with default settings. The reported data are per cell.

### Data Handling, Plots, and Statistics

Marvin was used for drawing, displaying, and characterizing chemical structures, version 23.11, 2023, ChemAxon (http://www.chemaxon.com). Protein schemes were drawn with the free package Illustrator for Biological Sequences.^70^ Data from predictions and experiments were treated and harmonized with Microsoft Excel 2016.

Plots and statistical tests were generated and performed with GraphPad Prism, version 9.0 (http://www.graphpad.com) and RStudio (vv 2022.12.0, https://posit.co/products/open-source/rstudio/) with the following packages: readxl, factoMineR, factoextra, ggplot2, tidyr, dplyr, reshape2, cowplot, ggrepel, rowr, openxlsx, matrixStats, and gridExtra.

## Supplementary Material



## Notes

MD simulations of VPS9 (Figure S1); pocket analysis (Figure S2); initial docking pose of MK4 compared with representative frames of MD simulations (Figure S3); *ALS2* expression in different tissues (Figure S4); procedure to establish a fibroblast cell line and mutation positions (Figure S5); mitochondria staining (Figure S6); mitochondria parameters on steady-state fibroblasts (Figure S7); mitochondria parameters on steady-state H_2_O_2_-treated fibroblasts (Figure S8); multiple comparisons of mitochondria parameters (Figure S9); TEM analysis (Figure S10); residues corresponding to the two identified subpockets (Table S1); residues of the VPS9 with contact frequency in MD simulation (Table S2); 3D mitochondrial parameters (descriptors) determined with Mitochondria Analyzer (Table S3); scripts to determine ligand interactions on MD trajectories with VMD (Supplementary Methods) (PDF)

## Abbreviations

ALS2: Amyotrophic Lateral Sclerosis 2
DH/PH: Dbl/Pleckstrin Homology domain
DRP1: Dynamin-1-like protein
HSPs: Hereditary Spastic Paraplegias
IAHSP: Infantile-onset Ascending Hereditary Spastic Paralysis
MK4: Menatetrenone
MORN: Membrane Occupation and Recognition Nexus
MD: Molecular Dynamics
MBD: Mean Branch Diameter
MFN2: Mitofusin 2
NMD: Nonsense-Mediated RNA Decay
PINK1: PTEN-induced putative kinase 1
Rab5: Ras-related protein Rab-5A
RAC1: Ras-related C3 botulinum toxin substrate 1
RLD: Regulator of Chromosome Condensation 1-like domain
RMSD: Root Mean Square Deviation
RMSF: Root Mean Square Fluctuation
ROS: Reactive Oxygen Species
TEM: Transmission Electron Microscopy
VMD: Visual Molecular Dynamics
VPS9: Vacuolar Protein Sorting 9 domain
