# Supplementary material for: MK4 Repositioning for IAHSP: Overcoming In Vivo Data Gaps through In Silico Refinement and In Vitro Validation
Source: ACS Chem Neurosci. 2026 Feb 9;17(6):1115–28. doi: 10.1021/acschemneuro.5c00601 (PMC13003441; doi:10.1021/acschemneuro.5c00601)
Supplement: Supplementary file 1 [file cn5c00601_si_001.pdf]

Supporting information

MK4 Repositioning for IAHSP: Overcoming *In Vivo* Data Gaps Through *In Silico* Refinement and *In Vitro* Validation

Matteo Rossi Sebastiano[1], Antonio Vicidomini[2], Serena Francisco[1], Verdiana Pullano[3], Paola Defilippi[1], Gabriele Baj[2], Fabrizia Cesca[4], Giulia Caron[1], and Giuseppe Ermondi[1]*

[1]University of Torino, Molecular Biotechnology and Health Sciences Department, Turin, Italy.

[2] University of Trieste, Life Science Department, Trieste, Italy

[3] University of Turin, Department of Neurosciences Rita Levi-Montalcini, Turin, Italy

[4] University of Padova, Department of Biomedical Sciences, Padova, Italy

*Corresponding author, Giuseppe Ermondi

List of contents

Supplementary Figures

Figure S1: MD simulations of VPS9.

Figure S2: Pocket analysis.

Figure S3: Initial docking pose of MK4 compared with representative frames of MD simulations.

Figure S4: *ALS2* expression in different tissues.

Figure S5: Procedure to establish a fibroblast cell line and mutation positions.

Figure S6: Mitochondria staining.

Figure S7: Mitochondria parameters on steady-state fibroblasts.

Figure S8: Mitochondria parameters on steady-state H_2O_2 treated fibroblasts.

Figure S9: Multiple comparisons of mitochondria parameters.

Figure S10: TEM analysis.

Supplementary Tables

Table S1: Residues corresponding to the two identified sub-pockets.

Table S2: Residues of the VPS9 with contact frequency in MD simulation.

Table S3: 3D mitochondrial parameters (descriptors) determined with Mitochondria Analyzer.

Supplementary Methods

Scripts to determine ligand interactions on MD trajectories with VMD

Supplementary Figures

Figure S1. MD simulations of VPS9. A) Root Mean Square Deviation and (B) Root Mean Square Fluctuation of WT and R1611W mutant VPS9 domain of Alsin models during the MD simulation. C) Clustering conformations from Molecular dynamics, 3D superposition of representative structures (C), frequency (D), and timeline progression (E).

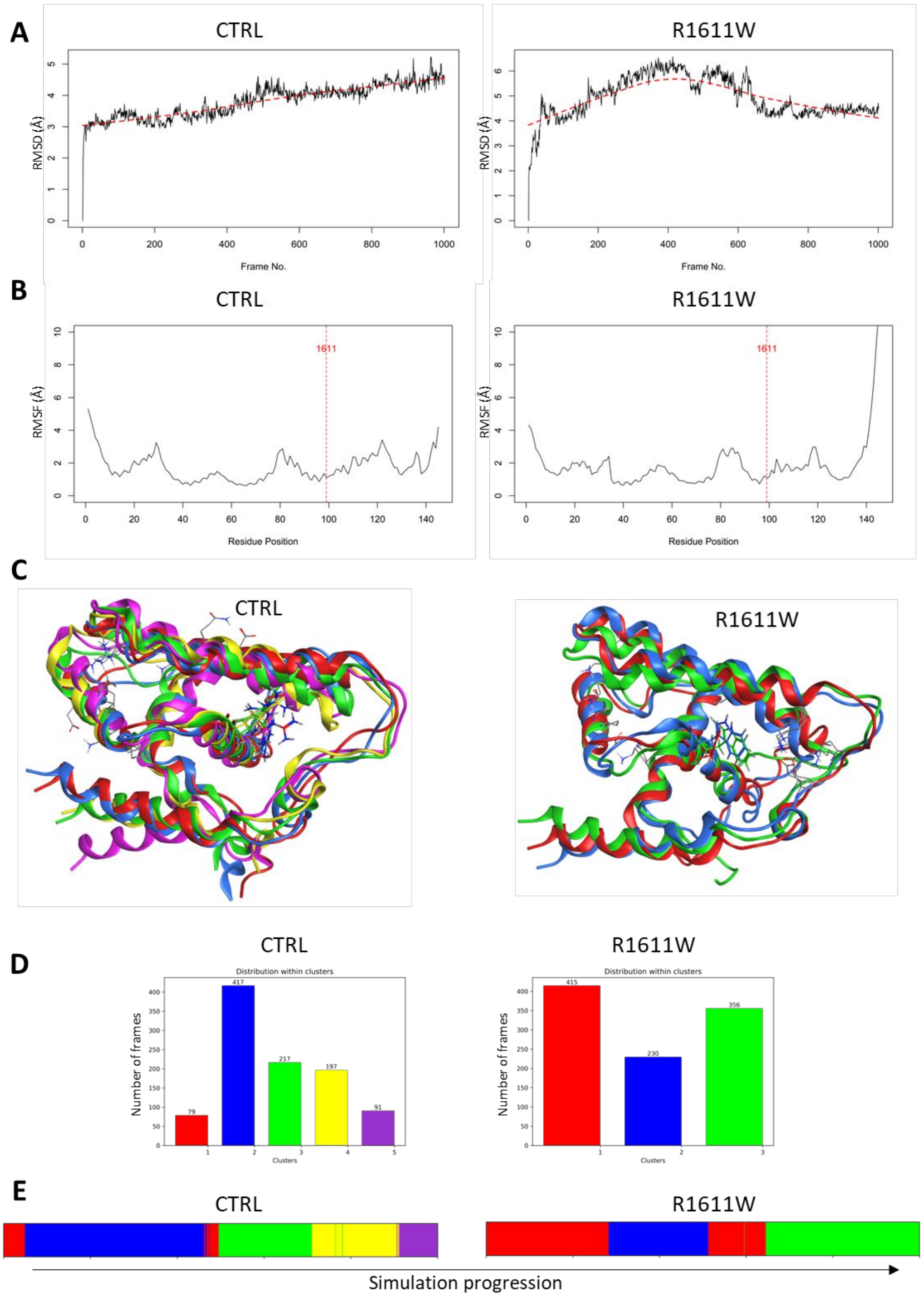

Figure S2. Pocket analysis. A) Apolar solvent-accessible Surface Area and Hydrophobicity Score in WT and R1611W VPS9 models subjected to 100ns MD. B) Non-smoothed curves tracking the Pocket volume on WT and R1611W mutant VPS9 domains from MD trajectories.

A

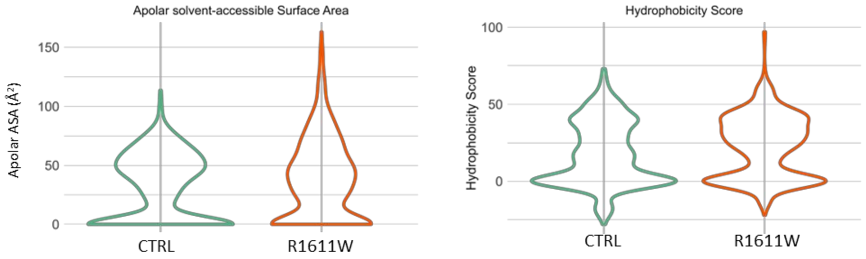
Apolar solvent-accessible Surface Area
Apolar ASA (Å²)
150
100
50
0
CTRL
R1611W
Hydrophobicity Score
Hydrophobicity Score
100
50
0
CTRL
R1611W

B

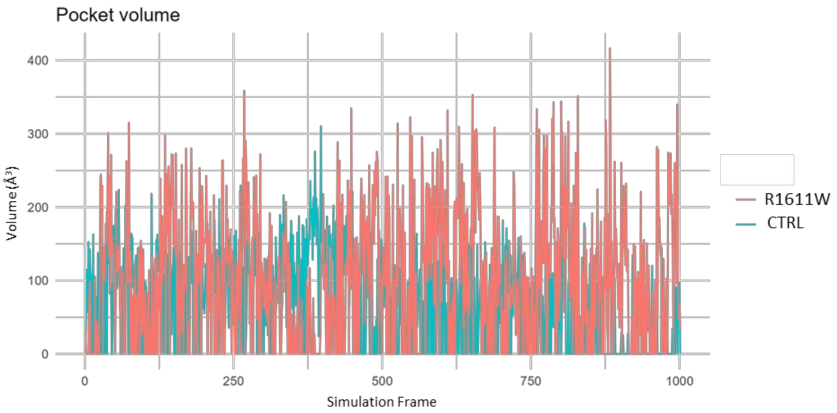
Pocket volume
Volume (Å³)
400
300
200
100
0
0
250
500
750
1000
Simulation Frame
R1611W
CTRL

Figure S3. Initial docking pose of MK4 compared with a representative frame of MD simulations.

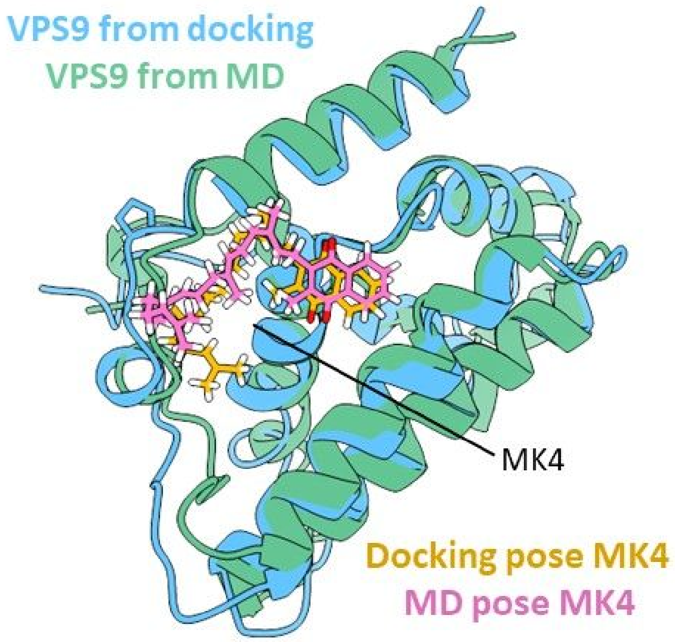

Figure S4. Scheme from the Human Protein Atlas featuring *ALS2* expression in different tissues.

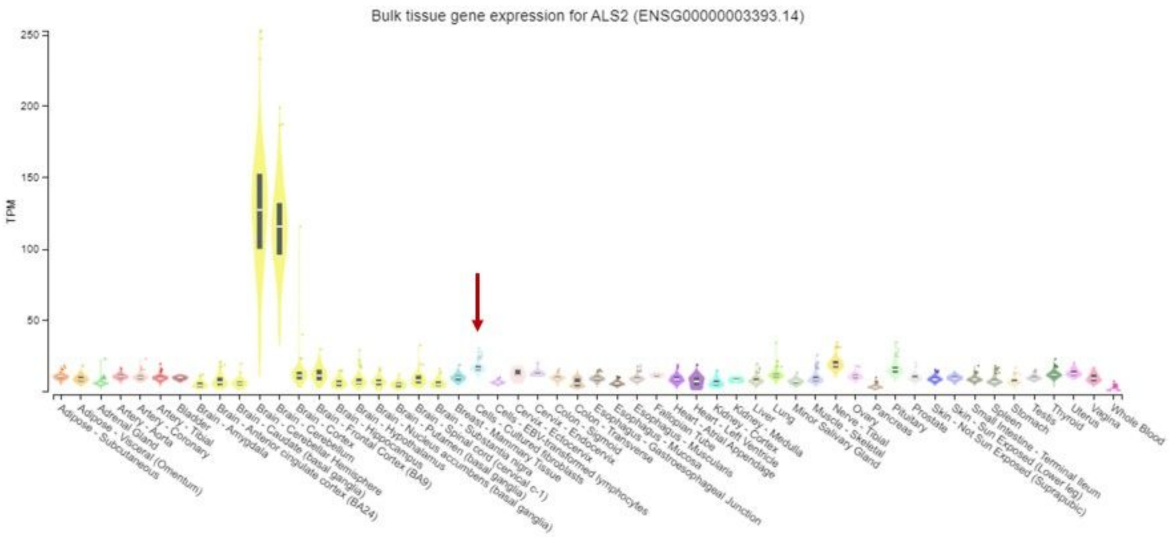

Figure S5. Patient-derived skin fibroblasts, Procedure to establish a fibroblast cell line and mutation positions. A) Schematic procedure for establishing a fibroblast cell line. B) Scheme of the domain within the coding region of Alsin and effect of the reported mutations from the Italian IAHSP patient. NMD border is the Nonsense Mediated mRNA decay predicted limit. *c.4831C>T, p.R1611W* is R1611W; *c.4368delG, p. K1457Sfs*14* is K1457*.

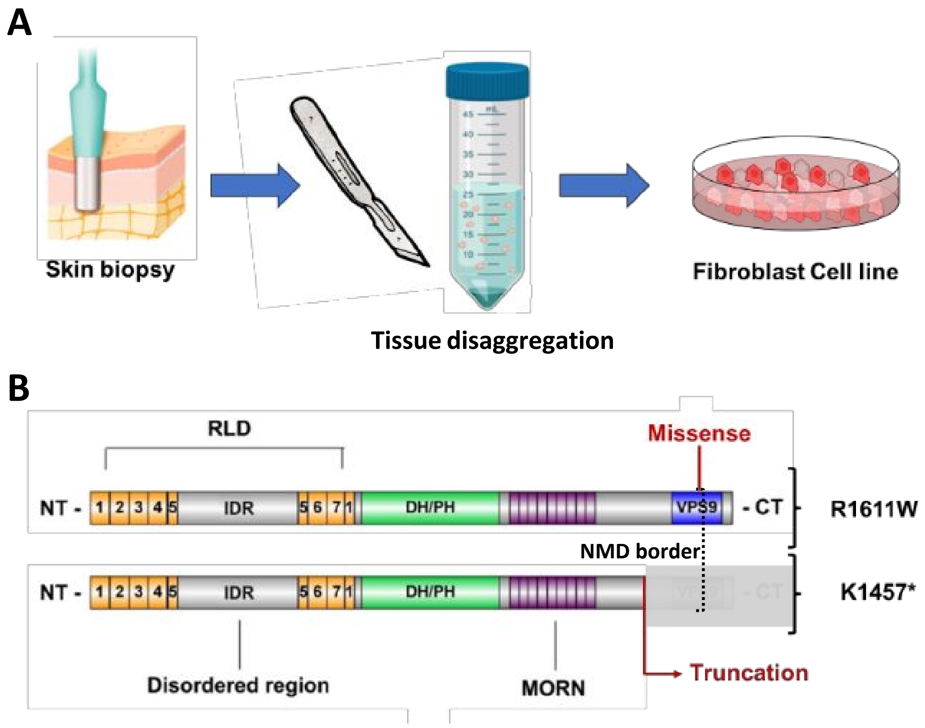

Figure S6. Mitochondria staining. A) Scheme for staining and batch mitochondria network analysis. B) Artifacts produced by 2D reconstruction and MitoTracker staining.

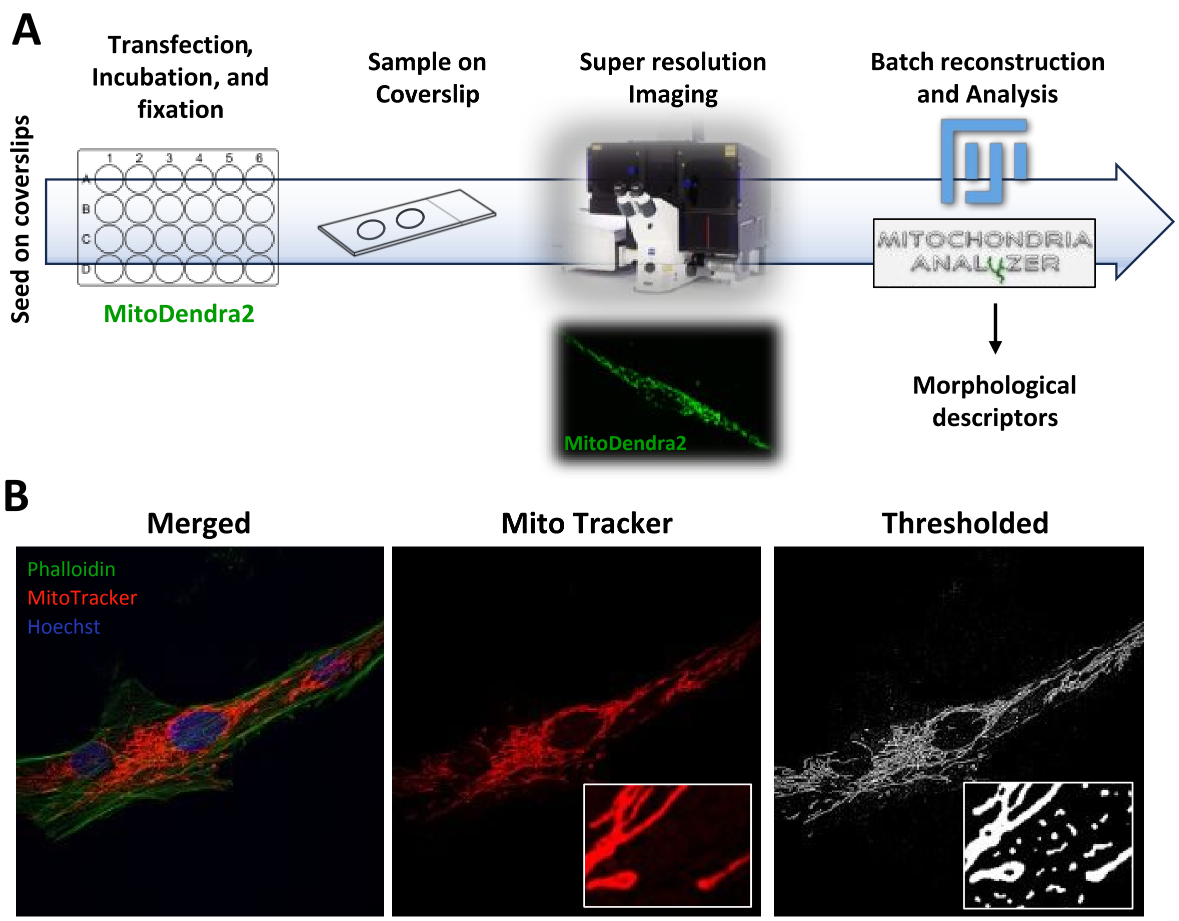

Figure S7. Analysis of mitochondria parameters on steady-state cells. Unpaired t-test (two-tailed); ** means 0.01 < p-value < 0.05*. Independent observations: $n = 10$ per group.

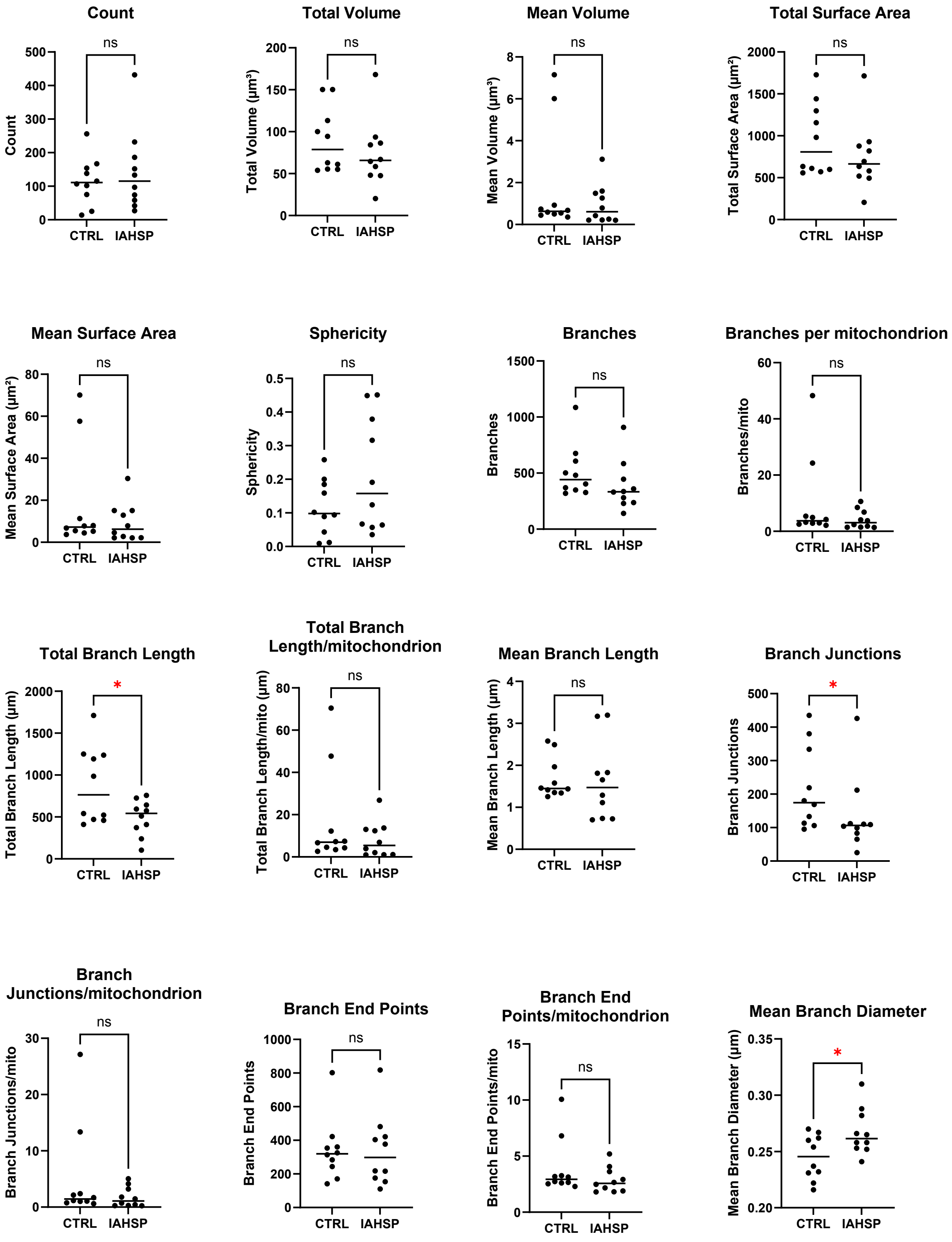

Figure S8. Analysis of mitochondria parameters of H_2O_2 treated fibroblasts. Unpaired t-test (two-tailed); ** means 0.01 < p-value < 0.05*. Independent observations: $n = 10$ per group.

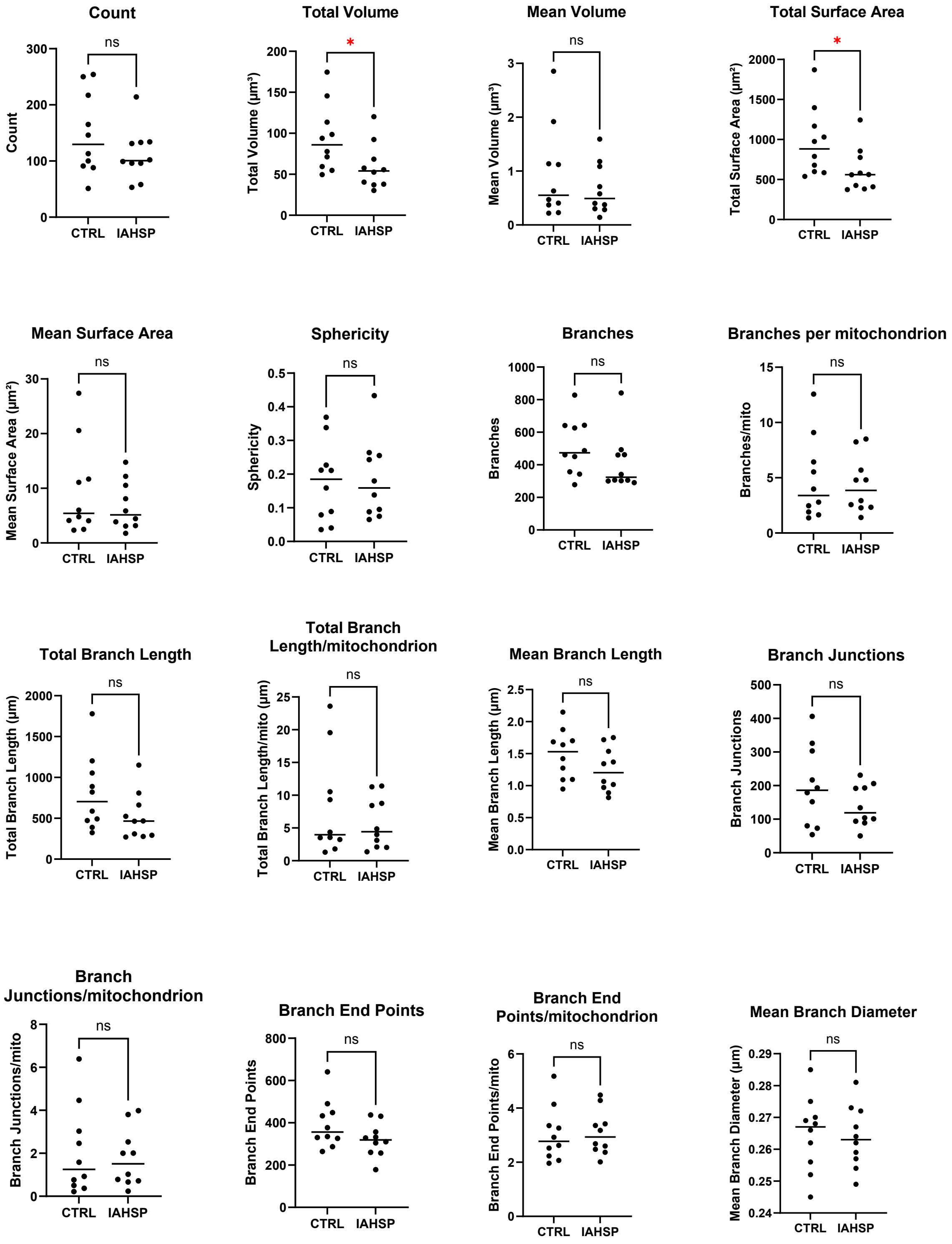

Figure S9. Analysis of Total Mitochondria volume, Total Mitochondria Surface Area and number of branch junctions performed by Two-Way ANOVA, followed by Tukey's multiple comparisons test; p values as indicated. Independent observations: n = 10 per group.

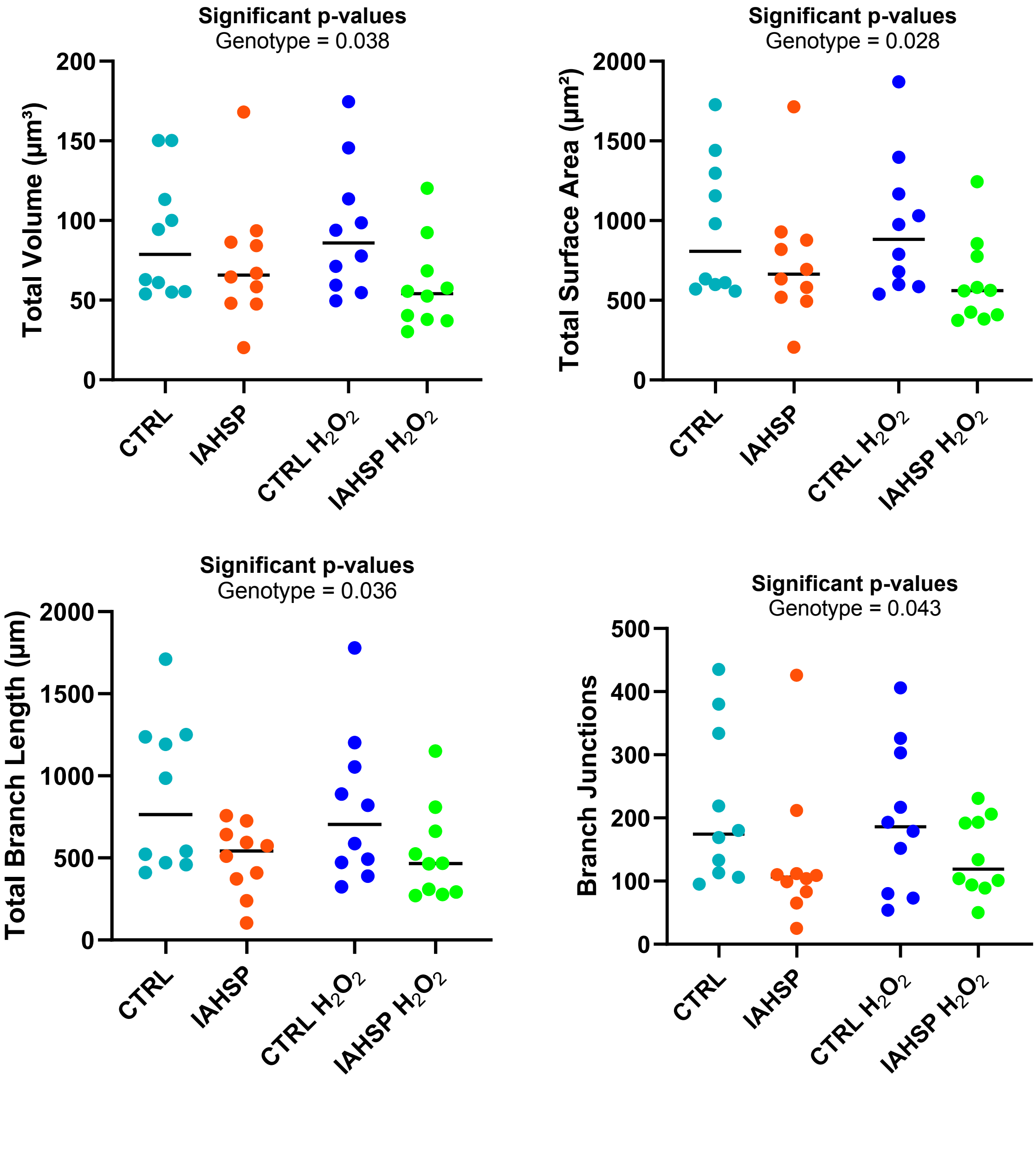

Figure S10. TEM analysis. A) Feret diameter and Aspect Ratio of mitochondria from TEM images suggests thicker mitochondria with altered shapes. B) Cristae parameters from TEM images. Significant differences are marked by * (**** , **, * p value < 0.001, 0.01, 0.05, respectively); Statistical analyses performed with Wilcoxon's t-test.

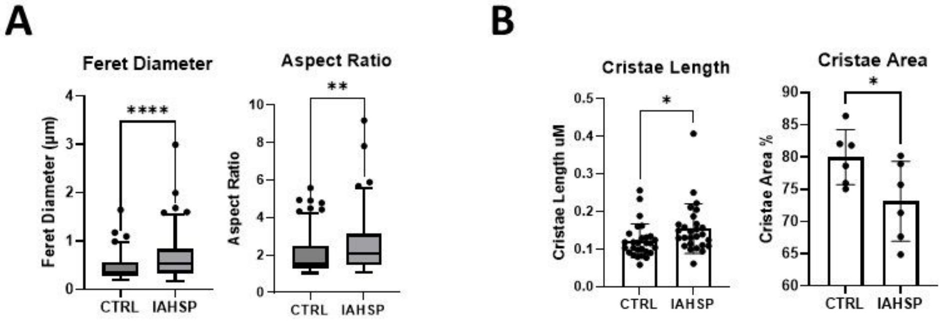

Supplementary Tables

Table S1. Residues corresponding to the two identified sub-pockets.

Sub pocket	Amino Acid
Sub pocket 1	1519
	1520
	1522
	1523
	1524
	1526
	1527
	1528
	1530
	1611
	1612
	1613
	1614
	1615
	1616
	1617
Sub pocket 2	1534
	1539
	1541
	1542
	1543
	1544
	1548

	1549
	1552
	1585
	1607

Table S2. Residues of the VPS9 with contact frequency in MD simulation.

Alsin Residue	Contacts count
1513	0
1514	0
1515	0
1516	0
1517	0
1518	0
1519	0
1520	0
1521	0
1522	212
1523	17
1524	0
1525	22
1526	285
1527	68
1528	1
1529	318
1530	391
1531	66
1532	178
1533	91
1534	266
1535	5
1536	9
1537	275
1538	0

1539	186
1540	1
1541	10
1542	0
1543	3
1544	0
1545	0
1546	0
1547	0
1548	0
1549	6
1550	0
1551	0
1552	73
1553	1
1554	0
1555	0
1556	3
1557	0
1558	0
1559	0
1560	3
1561	0
1562	0
1563	0
1564	0
1565	0
1566	0
1567	0
1568	0

1569	0
1570	0
1571	0
1572	0
1573	0
1574	0
1575	0
1576	0
1577	0
1578	0
1579	0
1580	0
1581	0
1582	0
1583	0
1584	0
1585	0
1586	0
1587	0
1588	0
1589	0
1590	0
1591	0
1592	0
1593	0
1594	0
1595	0
1596	0
1597	0
1598	0

1599	0
1600	0
1601	0
1602	6
1603	66
1604	7
1605	0
1606	98
1607	396
1608	3
1609	12
1610	397
1611	406
1612	13
1613	396
1614	4
1615	49
1616	0
1617	2
1618	0
1619	0
1620	0
1621	0
1622	0
1623	0
1624	0
1625	0
1626	0
1627	0
1628	0

1629	0
1630	0
1631	0
1632	0
1633	0
1634	0
1635	0
1636	0
1637	0
1638	0
1639	0
1640	0
1641	0
1642	0
1643	0
1644	0
1645	0
1646	0
1647	0
1648	9
1649	0
1650	0
1651	0
1652	0
1653	0
1654	0
1655	0
1656	0
1657	2

Table S3. 3D mitochondrial parameters (descriptors) determined with Mitochondria Analyzer.

**Type of parameter**	**Parameter**	**Description**
Morphological	Count	Number of mitochondria
	Total Volume	Sum of volume of all mitochondria
	Mean Volume	Total volume divided by count
	Total Surface Area	Sum of SA of all mitochondria
	Mean Surface Area	Total SA divided by count
	Sphericity	As objects become more spherical, the value approaches 1. expressed as a weighted mean of the image, by weighting each object's sphericity to its volume
Network	Branches	Total number of branches
	Total Branch Length	Sum of length of all branches
	Mean Branch Length	Total branch length divided by number of branches
	Branch Junctions	Number junctions within all skeletons in image. Junctions are points where 2 or more branches meet
	Branch End Points	Total number of endpoint, which are where branches end without connecting to another branch
	Mean Branch Diameter	The mean diameter across all objects, by computing a euclidean distance map and using their skeletons to identify the long axis from which to measure diameter across

<u>Supplementary Methods:</u>

Script 1: identification of contacts using VMD and tk console

```
set num_frames [molinfo top get numframes]
for {set frame 0} {$frame < $num_frames} {incr frame} {
   # Change to the current frame
   molinfo top set frame $frame
   # Select the ligand and nearby atoms
   set ligand [atomselect top "fragment 1"]
   set nearby [atomselect top "within 4.5 of fragment 1"]

   # Get unique residue IDs
   set unique_data [lsort -unique [$nearby get resid]]

   # Optionally, do something with unique_data
   puts "Frame $frame: Unique residues - $unique_data"
}
```

Script 2: Identification of π-contacts with VMD and tk console

```
set num_frames [molinfo top get numframes]

for {set frame 0} {$frame < $num_frames} {incr frame} {

    # Change to the current frame

    molinfo top set frame $frame


    # Select the ligand atoms by their index

    set ligand [atomselect top "index 2355 2365 2364 2375 2379 2380 2376 2366 2370 2361"]


    # Calculate the centroid of the ligand

    set ligand_centroid [measure center $ligand]


    # Select atoms of TRP, PHE, TYR, and HIS side chains within 4.5 Å of any ligand atom, excluding the backbone

    set nearby [atomselect top "resname TRP PHE TYR HIS and within 4.5 of index 2355 2365 2364 2375 2379 2380 2376 2366 2370 2361 and not backbone"]


    # Get unique residue IDs (resid) and names (resname)

    set unique_data [lsort -unique [$nearby get {resid resname}]]
```

```
# Loop through each unique residue
foreach resinfo $unique_data {
    # Extract residue id and name
    lassign $resinfo resid resname

    # Select the sidechain atoms for the current residue
    set sidechain [atomselect top "resid $resid and resname $resname and not backbone"]

    # Calculate the centroid of the sidechain
    set sidechain_centroid [measure center $sidechain]

    # Calculate the distance between the ligand and sidechain centroids
    set distance [veclength [vecsub $ligand_centroid $sidechain_centroid]]
    # If the distance is less than or equal to 4.5 Å, report the residue
    if {$distance <= 6} {
        puts "Frame $frame: $resname $resid sidechain centroid is within 6 Å of the ligand"
    }
```

```
    }
}
```
